# A Dual‐Modal Wearable Pulse Detection System Integrated with Deep Learning for High‐Accuracy and Low‐Power Sleep Apnea Monitoring

**DOI:** 10.1002/advs.202501750

**Published:** 2025-04-29

**Authors:** Jia Wang, Jiangtao Xue, Yang Zou, Yuxin Ma, Junhan Xu, Yanming Li, Fei Deng, Yiqian Wang, Kai Xing, Zhou Li, Tong Zou

**Affiliations:** ^1^ Department of Cardiology Beijing Hospital National Center of Gerontology Institute of Geriatric Medicine Chinese Academy of Medical Sciences & Peking Union Medical College Beijing 100730 China; ^2^ Beijing Institute of Nanoenergy and Nanosystems Chinese Academy of Sciences Beijing 101400 China; ^3^ School of Medical Technology Beijing Institute of Technology Beijing 100081 China; ^4^ School of Computer Science and Suzhou Institute for Advanced Research University of Science and Technology of China Hefei Anhui 230026 China; ^5^ Department of Cardiology Beijing Hospital National Center of Gerontology Institute of Geriatric Medicine Chinese Academy of Medical Sciences & Peking University Fifth School of Clinical Medicine Beijing 100730 China; ^6^ Department of Pulmonary and Critical Care Medcine Beijing Hospital National Center of Gerontology Institute of Geriatric Medicine Chinese Academy of Medical Sciences Beijing 100730 China; ^7^ School of Nanoscience and Technology University of Chinese Academy of Sciences Beijing 100049 China

**Keywords:** deep learning, photoplethysmography, piezoelectric nanogenerator, pulse detection, sleep apnea monitoring

## Abstract

Despite being a serious health condition that significantly increases cardiovascular and metabolic disease risks, sleep apnea syndrome (SAS) remains largely underdiagnosed. While polysomnography (PSG) remains the gold standard for diagnosis, its clinical application is limited by high costs, complex setup requirements, and sleep quality interference. Although wearable devices using photoplethysmography (PPG) have shown promise in SAS detection, their continuous operation demands substantial power consumption, hindering long‐term monitoring capabilities. Here, a dual‐modal wearable system is presented integrating a piezoelectric nanogenerator (PENG) and PPG sensor with a biomimetic fingertip structure for SAS detection. A two‐stage detection strategy is adopted where the self‐powered PENG performs continuous preliminary screening, activating the PPG sensor only when suspicious events are detected. Combined with a Vision Transformer‐based deep learning model, the high‐accuracy configuration achieves 99.59% accuracy, while the low‐power two‐stage approach maintained 94.95% accuracy. This dual‐modal wearable pulse detection system provides a practical solution for long‐term SAS monitoring, overcoming the limitations of traditional PSG while maintaining high detection accuracy. The system's versatility in both home and clinical settings offers the potential for improving early detection rates and treatment outcomes for SAS patients.

## Graphical abstract

A dual‐modal wearable pulse detection system is developed for sleep apnea monitoring by integrating a self‐powered PENG and PPG sensor with a biomimetic fingertip structure. Through a two‐stage detection strategy, continuous PPW monitoring and on‐demand PPG detection achieve high‐accuracy sleep apnea recognition while maintaining low power consumption. The deep learning model enables accurate identification of sleep apnea events, providing a practical solution for both home screening and clinical diagnosis.

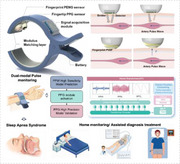

## 1 Introduction

Sleep Apnea Syndrome (SAS) is a prevalent sleep‐breathing disorder, classified into obstructive sleep apnea (OSA), central sleep apnea (CSA), and mixed sleep apnea (MSA), with OSA being the most common subtype. Globally, OSA affects over 24% of the population, a prevalence that continues to rise annually. Alarmingly, >80% of patients remain undiagnosed and untreated.^[^ ^1^, ^2^ ^]^ SAS not only significantly reduces quality of life, but is also strongly associated with the development of cardiovascular diseases such as hypertension, arrhythmia, and heart failure, as well as metabolic diseases, significantly increasing the risk of death and disability.^[^ ^3^ ^]^ Consequently, early screening and diagnosis of SAS are of critical clinical importance for reducing the burden of associated diseases. Currently, polysomnography (PSG)^[^ ^4^ ^]^ is the gold standard for diagnosing SAS. By synchronously recording physiological parameters such as electroencephalography, electrocardiography, electromyography, electrooculography, nasal airflow, respiratory effort, and blood oxygen saturation, PSG provides a comprehensive assessment of sleep structure and respiratory events. However, several limitations hinder its widespread application:^[^ ^5^ ^]^ PSG requires a specialized sleep laboratory environment and skilled personnel, involves costly equipment and testing procedures, and often compromises patients’ sleep quality due to the discomfort of multi‐channel sensor attachments, potentially leading to inaccurate results. These challenges restrict the feasibility of PSG for large‐scale population screening and long‐term dynamic monitoring, necessitating the exploration of more portable and user‐friendly alternatives.

In recent years, rapid advancements in microelectronics and artificial intelligence (AI) algorithms have highlighted the potential of wearable devices for sleep monitoring.^[^ ^6^, ^7^, ^8^, ^9^, ^10^, ^11^ ^]^ In particular, photoplethysmography (PPG) has gained significant attention due to its non‐invasive and portability).^[^ ^12^, ^13^, ^14^, ^15^, ^16^ ^]^ PPG detects periodic changes in blood volume and blood oxygen saturation, reflecting local hemodynamic characteristics and oxygenation status. Numerous studies have demonstrated the utility of PPG signals in detecting respiratory events associated with sleep apnea. For instance, Jesús et al.^[^ ^17^ ^]^ achieved an 86.67% accuracy in distinguishing between apneic and non‐apneic events by extracting pulse rate variability (PRV) features from PPG signals. Similarly, Liu et al.^[^ ^18^ ^]^ developed a multi‐task learning model (1D‐MMResSNet) that enhanced the ability to extract subtle differences in PPG data, achieving detection accuracy, sensitivity, and specificity of 95.65%, 88.89%, and 97.30%, respectively. Particularly noteworthy is PPG's capacity for real‐time monitoring of dynamic blood oxygen saturation (SpO₂) changes, providing a critical indicator for non‐invasive identification of apnea events.^[^ ^19^, ^20^, ^21^, ^22^ ^]^ However, PPG sensors rely on active light sources for detection, resulting in high power consumption, which poses challenges for long‐term continuous monitoring.^[^ ^23^, ^24^, ^25^ ^]^

Piezoelectric nanogenerators (PENG) offer unique advantages in physiological signals monitoring due to their high sensitivity to mechanical signals and self‐powered properties.^[^ ^26^, ^27^, ^28^, ^29^ ^]^ By directly converting minor mechanical deformations into electrical signals, PENG reduces the overall power consumption of signals acquisition systems, making it particularly suitable for long‐term continuous monitoring applications. In the field of cardiovascular health, PENG has demonstrated high sensitivity and specificity for atrial fibrillation screening and blood pressure assessment.^[^ ^27^ ^]^ Regarding apnea detection, Panda et al.^[^ ^30^ ^]^ utilized highly sensitive PENG devices to identify apnea events from snoring signals characteristics, enabling real‐time monitoring. Lin et al.^[^ ^31^ ^]^ monitored thoracoabdominal respiratory movements using piezoelectric sensors to successfully distinguish CSA from OSA. Despite these advances, research has yet to explore the use of piezoelectric sensors for pulse pressure wave monitoring in sleep apnea detection. Unlike PPG, which measures volumetric changes in blood via optical principles, PENG captures dynamic changes in vascular pressure through the stress‐strain response of blood vessel walls.^[^ ^32^ ^]^ This mechanical signals‐based detection mechanism provides instantaneous feedback by directly measuring vascular wall deformations, bypassing the lag associated with blood oxygen saturation changes. Thus, PENG may offer a novel pathway for the early identification of apnea events.

Building on this research landscape, we propose an innovative dual‐modal wearable pulse detection system. The hardware design incorporates biomimetic principles, employing a fingertip‐like structure to simulate the palpation process of traditional Chinese medicine, ensuring stable contact between sensors and the skin. The system integrates PENG and PPG sensors, leveraging their respective strengths through a two‐stage detection strategy. In the first stage, PENG's low‐power advantage facilitates 24‐h continuous prescreening. Upon detecting suspicious events, the system activates PPG for precise detection in the second stage. This strategy overcomes the limitations of single‐sensor approaches while optimizing the trade‐off between power consumption and detection accuracy. At the algorithmic level, this study is based on the deep learning model of Vision Transformer,^[^ ^33^ ^]^ which can effectively extract and analyze the high‐dimensional features of sensor signals. Compared to traditional low‐dimensional statistical feature‐based methods, this model achieves superior performance, with a high‐accuracy configuration reaching 99.59% accuracy and a low‐power configuration maintaining 94.95% accuracy in identifying apnea events. The wrist‐worn dual‐modal sensing device offers a novel technological pathway for SAS detection, addressing the limitations of conventional PSG while enabling low‐power, high‐accuracy, and portable long‐term dynamic monitoring. The system holds broad applicability for home screening, clinical diagnosis, and treatment evaluation, promising to improve the early detection and management of SAS significantly. A comprehensive comparison between our proposed system and existing SAS detection methods is provided in Table S1 (Supporting Information), highlighting the advantages of our approach in terms of detection principles, algorithms, recognition accuracy, and power consumption.

## 2 Results and Discussion

### 2.1 Design of Dual‐Modal Wearable Pulse Detection System

**Figure 1:**
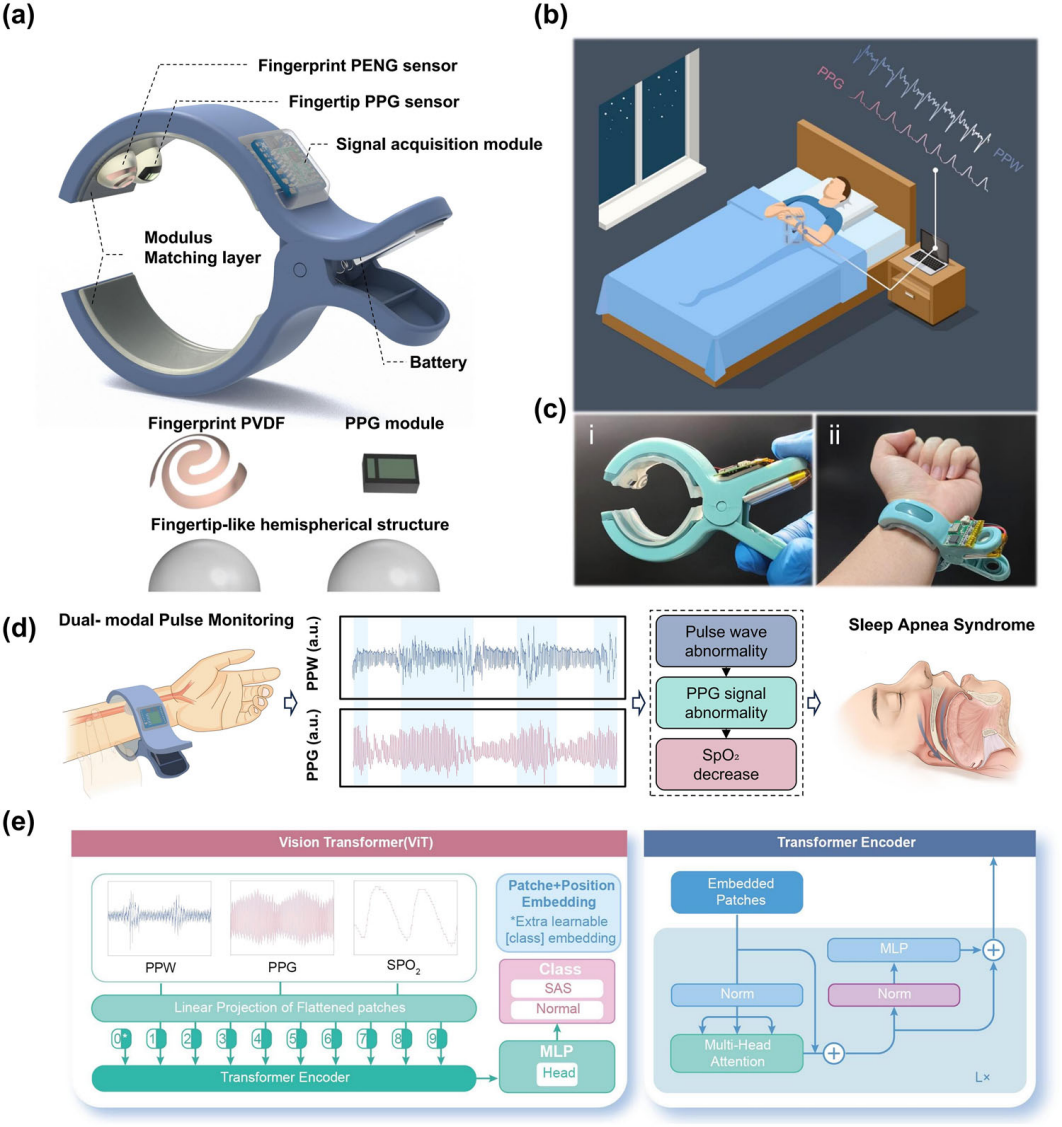
A dual‐modal wearable pulse detection system integrated with deep learning for sleep apnea monitoring. a) Schematic diagram of the dual‐modal wearable pulse detection system, along with an exploded view of the sensor module, showing the C‐shaped clamp structure, elastic layer, and integrated PENG and PPG sensors. b) Long‐term continuous monitoring of pulse during home sleep, including a schematic representation of PPW and PPG signals during normal breathing and apnea events. c) Physical photograph of the dual‐modal wearable pulse detection system as worn on a subject's wrist, demonstrating its compact and portable design. d) Fundamental principles and processes of the system for monitoring sleep apnea, illustrating the two‐stage detection strategy where PENG performs continuous screening and PPG activates only when necessary. e) Vision Transformer (ViT) deep learning model used for classifying sleep apnea data, showing the patching process, attention mechanism, and classification output.

Traditional Chinese medicine (TCM) pulse diagnosis has a long history and possesses unique advantages and value in clinical practice. TCM Practitioners typically apply pressure with their fingertips to specific points on the wrist, using their keen sense of touch to perceive the characteristics of the pulse intuitively and sensitively, such as its strength, speed, and rhythm. Inspired by TCM pulse palpation, a wrist‐worn dual‐modal pulse monitoring device has been designed, as shown in **Figure** **1**a. The system can be divided into four parts: the force application device, silicone elastic layer, sensing module, and communication module. The force application device is designed as a C‐shaped clamp, with the curvature based on ergonomic principles. Through structural design, the elastic potential energy of the spring can be converted into pressure applied to the wrist. The spring mechanism in the clip serves as an adaptive tension regulator, determining the applied pressure. We tested individuals with different wrist sizes and ultimately selected an appropriate spring wire diameter and coil diameter to ensure appropriate pressure without causing discomfort across diverse populations. The silicone elastic layer is constructed from Ecoflex‐30 and consists of two distinct segments: a modulus‐matching layer and a hemispherical structure designed to simulate the fingertip. Since the clip is composed of rigid plastic, extended use may cause skin irritation. To alleviate this issue, a modulus‐matching silicone elastomer layer with mechanical properties similar to human skin was introduced between the C‐shaped clip and the skin surface. This elastic silicone layer prevents stress concentration at the hard‐shell–skin interface, further enhancing long‐term wear comfort. The hemispherical structure is designed based on the morphology of a fingertip, aiming to simulate the pressing process of the fingertip during pulse diagnosis, thereby achieving stress concentration at the radial artery for high‐sensitivity monitoring. The sensing module is divided into two parts, one of which is the pulse pressure sensing unit that uses a fingerprint‐like piezoelectric sensor. The fingerprint‐like structural design allows the polyvinylidene fluoride (PVDF) film to conform closely to the spherical protrusions, and enhances the sensitivity to the changes in pulse pressure. The other part is the PPG unit, which embeds the PPG probe into the surface of the fingertip‐like hemispherical structure. The fingertip‐like hemispherical structure and the force application device ensure close contact between the sensor and the skin, reducing motion artifacts and other noise while improving signals stability. The communication module includes a lithium battery and a signals acquisition circuit, enabling continuous collection and wireless transmission of the pulse pressure wave (PPW) and PPG signals.

Through a two‐stage detection strategy, the dual‐modal wearable pulse detection system can effectively acquire real‐time continuous PPW and the necessary PPG signals to reflect the occurrence of sleep apnea events (Figure 1b). Figure 1c shows the physical photograph of the entire monitoring system, which can be conveniently and comfortably worn on the radial artery site of the subject. The fundamental principles and processes of the system for monitoring sleep apnea are illustrated in Figure 1d. By utilizing the advantages of low‐power PENG, continuous high‐sensitivity monitoring of pulse pressure can be achieved. When abnormalities in the pulse pressure wave are detected, the PPG module is activated to perform a precise assessment of sleep apnea syndrome through the analysis of indicators such as blood oxygen levels. This two‐stage detection strategy not only enhances monitoring accuracy but also reduces the overall power consumption of the wearable monitoring system. The system integrates a PENG sensor and a PPG sensor (MAX30102), transmitting signals via a low‐power Bluetooth module (E104_BT5005A). Compared to the PPG module, the self‐powered PENG sensor operates with negligible power consumption. Meanwhile, the PPG sensor, serving as a high‐precision oxygen saturation (SpO₂) and heart rate monitor, has its continuous power consumption primarily determined by its LED (∼34 mW). The Bluetooth module and MCU operates at ∼40 mW in continuous mode. Due to the two‐stage detection strategy, and depending on the frequency of sleep respiratory events, the PPG sensor is activated ∼15 times per hour. Consequently, the total estimated power consumption of the system is ≈57 mW—compared to 74 mW when using PPG continuously—resulting in a ∼23% reduction in power consumption. Furthermore, a Vision Transformer (ViT) deep learning model is employed to handle high‐dimensional feature extraction and classification of sleep apnea data, outperforming traditional low‐dimensional approaches. The model processes input sensing signals—PPW, PPG, and SpO₂—by first projecting them into flattened patches, which are then augmented with positional embeddings and an additional learnable class embedding. These patches are passed through the Transformer encoder, which incorporates multi‐head attention, normalization, and MLP layers to capture complex patterns and temporal relationships. The model's final MLP head outputs the classification result, distinguishing between “SAS” (sleep apnea syndrome) and “Normal” states. This approach enables the ViT model to effectively learn and classify the intricate patterns associated with sleep apnea detection, as shown in Figure 1e.

### 2.2 Characterization of the Dual‐Modal Wearable Pulse Detection System

**Figure 2:**
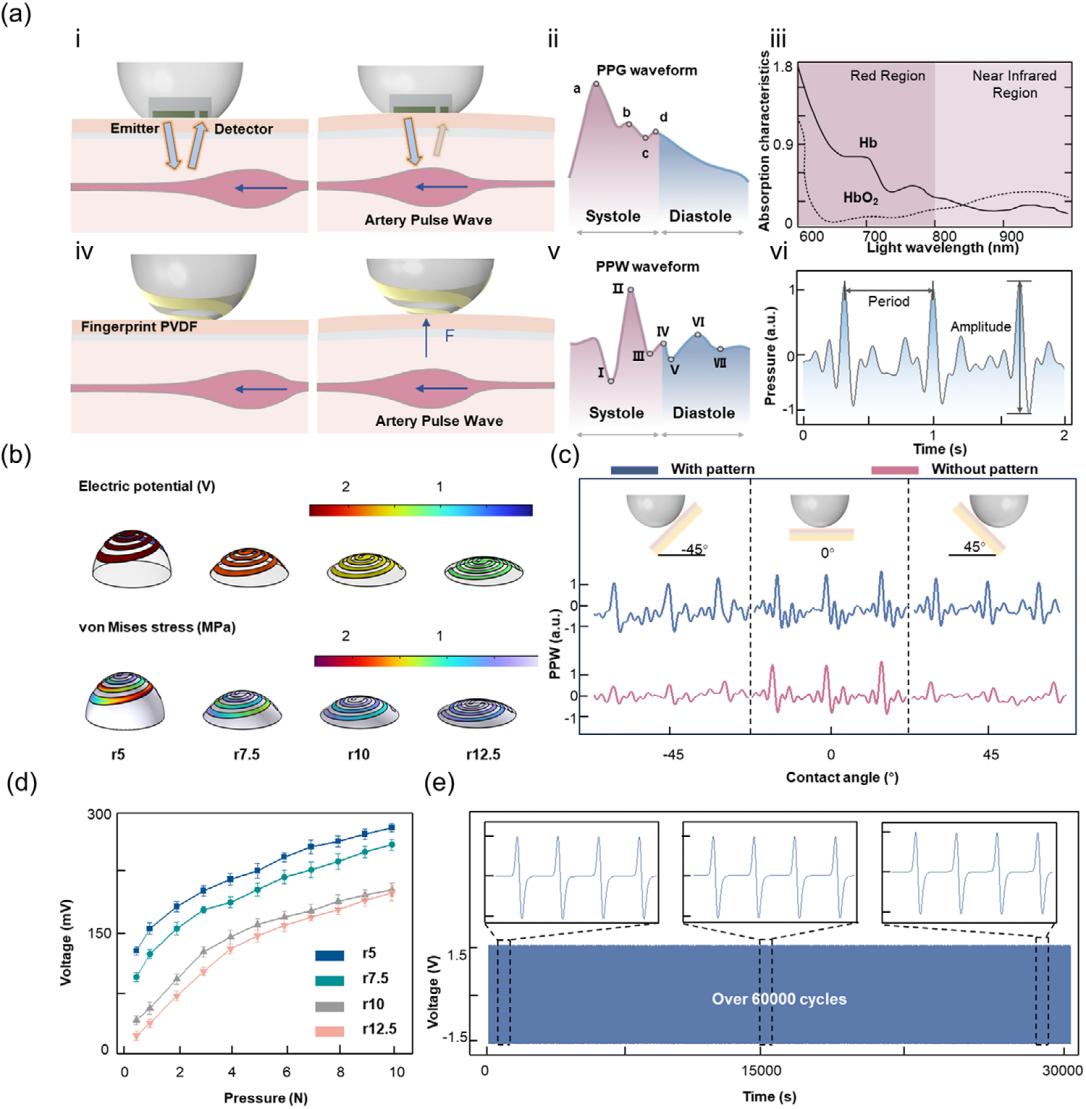
Principle and characterization of the fingertip‐shaped pulse monitoring sensor. a) PPG and PPW sensor module pulse monitoring principles: (i) Schematic diagram of PPG pulse monitoring principles. (ii) Schematic representation of PPG waveform. (iii) Schematic illustration of PPG monitoring blood oxygen principles. (iv) Schematic diagram of PPW pulse monitoring principles. (v) Schematic representation of PPW waveform. (vi) Schematic illustration of the amplitude and period of the PPW waveform. b) Finite element simulation results of the mechanical and electrical properties of arc structures with fingerprint‐like PVDF at different curvature radii under the same pressure conditions. c) Output comparison between sensors with and without fingerprint‐like PVDF pattern structures at different angles. d) Force‐electric response relationship measured in the mechanical characterization of arc structures with different curvature radii. e) Output curve of the PPW sensor module during more than 60 000 pressure tests.

The dual‐modal wearable pulse detection system combines the principles of TCM pulse diagnosis with two methods of pulse monitoring, achieving comprehensive and accurate monitoring of pulse abnormalities. The monitoring principles are illustrated in **Figure** **2**a. First is the schematic diagram of the PPG principle (Figure 2a‐i). The PPG module consists of an emitter and a detector, which are placed closely against the skin surface to capture the changes in arterial blood volume. Variations in the arterial blood volume lead to changes in the amount of light absorbed by the tissue, generating detectable signals. Figure 2a‐ii shows a single waveform of the PPG signals, which includes the systolic and diastolic phases. Different characteristic points of the waveform reflect various stages of the pulse wave. Point a represents the main wave, reflecting the maximum pressure and volume within the artery, corresponding to the highest pressure during the cardiac contraction phase. Point b indicates the tidal wave, which is the first trough and peak after the main wave, reflecting the backflow phenomenon when the aortic valve closes. Point c is the descending inflection, representing the local minimum following the tidal wave. Its position and depth can reflect the elasticity and resistance status of the blood vessels. Point d represents the bidirectional wave, indicating blood returning to the left ventricle.^[^ ^34^, ^35^, ^36^, ^37^ ^]^ Figure 2a‐iii illustrates the principle of the PPG module measuring blood oxygen saturation. As shown, HbO_2_ and Hb exhibit different optical absorption characteristics; for instance, Hb has a higher absorption coefficient in the range of 600 to 800 nm, while HbO_2_ has a higher absorption coefficient in the range of 800 to 1000 nm. By utilizing this characteristic and combining it with a computational model, the ratios of these two can be detected separately, allowing the determination of SpO₂. The schematic diagram of the PPW module is shown in Figure 2a‐iv. By utilizing the high sensitivity characteristics of fingerprint PVDF, and under pressure applied by the fingertip‐like hemispherical structure, it can reflect slight pressure changes on the vascular surface during the pulse process, thus recording the PPW signals. Its individual waveform (Figure 2a‐v) also records different stages of the pulse process. Point I represents the early phase of ventricular contraction, while point II marks the end of the main wave and indicates the beginning of ventricular relaxation. Point III indicates the backflow in the aorta during the temporary closure of the mitral valve. Point IV represents the descending trough between the main wave and the tidal wave. Point V corresponds to the descending portion of the slow wave, which is related to arterial elasticity and resistance. Point VI reflects the time taken for the pulse wave to propagate to the end, indicating the resistance state of the small arteries, while point VII reflects the microcirculation response and terminal pressure.^[^ ^32^, ^38^, ^39^ ^]^ Due to the high sensitivity characteristics and good frequency response of Fingerprint PVDF, it performs well in monitoring the magnitude and period of vascular contraction (Figure 2a‐vi), making it significant for monitoring pulse wave amplitude (PWA) and heart rate variability (HRV). To be precise, our system directly measures pulse rate variability (PRV) rather than HRV, as the latter requires ECG recordings. However, substantial evidence supports that PRV derived from pulse signals strongly correlates with HRV under controlled conditions, making it a valuable surrogate for assessing autonomic function during sleep.^[^ ^40^, ^41^ ^]^ While PRV and HRV may show some divergence during significant hemodynamic changes, this divergence itself provides additional diagnostic information during sleep apnea events by capturing the vascular component of the autonomic response.^[^ ^42^ ^]^ To demonstrate that the highly sensitive PENG sensor achieves consistent results with the PPG sensor in PRV monitoring, both sensors were simultaneously placed on a subject's radial artery to record pulse waveforms for 1 min (Figure S1, Supporting Information). The results showed high morphological consistency between the two waveforms. Further statistical analysis of adjacent peaks in both waveforms revealed a strong linear correlation (R^2^ = 0.9936). Therefore, the PENG sensor exhibits performance comparable to that of the high‐precision PPG sensor in PRV monitoring.

The performance of the fingertip‐like hemispherical structure and fingerprint PVDF was characterized. First, finite element simulation software COMSOL Multiphysics was used to perform mechanical and electrical field simulations on protruding structures with different curvature radii (Figure 2b). Here, r5, r7.5, r10, and r12.5 represent arc structures with curvature radii of 5, 7.5, 10, and 12.5 mm, respectively. By comparing the structures, it was found that when the curvature radius is 5, the stress experienced by the fingerprint‐like PVDF attached to it is the greatest under the same external force conditions. Therefore, the surface potential under the same conditions is the highest, leading to the selection of the fingertip‐like hemispherical structure with a curvature radius of 5 mm. Additionally, we created arc structures with different curvatures to further validate this conclusion. In Figure S2 (Supporting Information), these different arc structures were used to test the radial artery of the same subject. Compared to other curvature radii, the arc structure with a curvature radius of 5 mm not only exhibits distinct characteristic points in the PPW signals but also has the highest amplitude, thereby confirming the results of the theoretical calculations. At the same time, we verified the advantages of using fingerprint PVDF by applying both ordinary PVDF film and fingerprint PVDF to the arc structure and using them together for pulse testing. As shown in Figure 2c, although the amplitude of the pulse wave is nearly identical when the sensors with pattern and without pattern are in direct vertical contact with the skin, represented by the 0° position in the figure, a significant decrease in output occurs in the sensor without pattern when the applied force is not vertical to the skin. In contrast, the reduction in output for the sensor with pattern is not significant. This demonstrates the versatility of using fingerprint PVDF for different contact angles. Subsequently, the mechanical responsiveness of the sensor module was tested. As shown in Figure 2d, the fingertip‐like hemispherical structure with a curvature radius of 5 mm exhibits superior mechanical response capability compared to structures with other curvature radii, further confirming the effectiveness of the structure. The normal heart rate during sleep is ≈60–100 bpm. To verify that the PENG sensor maintains good output stability within this range, four external excitation frequencies from 0.5 to 2 Hz were set, covering the pulse frequency range corresponding to sleep heart rates. Testing results in Figure S3 (Supporting Information) showed that, except for 0.5 Hz, there is no obvious difference in the output amplitude of the PENG at other frequencies, confirming the sensor's stable amplitude output characteristic across the heart rate range. Additionally, to ensure that the PENG can accurately capture pulse waveform variations, we further characterized its dynamic response performance. The PENG sensor was placed on a vibration table and subjected to 50 Hz external excitation. Test results demonstrated that under this excitation frequency, the response and recovery time of the PENG was 50 ms (Figure S4, Supporting Information). Although this does not represent its ultimate fast‐response limit, the achieved performance is fully adequate for instantaneously tracking pulse wave changes.

A long‐duration fatigue test on the PPW module was also conducted. The experimental results indicated (Figure 2e) that after more than 60 000 cycles of elastic compression testing through a motorized vertical test stand, the output performance of the PENG sensor remained nearly unchanged, demonstrating its excellent stability for long‐term continuous monitoring. To further validate the long‐term monitoring capability of the PENG sensor, we examined its output variations under different external conditions. First, we conducted environmental tests at five humidity levels (20% to 100%). Thanks to the excellent hydrophobicity of the PTFE film encapsulation layer, the PENG sensor's output remained nearly unchanged across humidity gradients (Figure S5a, Supporting Information). Next, we tested five temperature gradients (20–40 °C) near body temperature. Similarly, the sensor's output showed minimal variations under the same excitation, as temperatures in this range do not significantly alter the piezoelectric coefficients of the material, ensuring stable performance (Figure S5b, Supporting Information). Meanwhile, to evaluate the impact of storage time on the PENG sensor's performance, we conducted comparative tests between a freshly prepared sensor and one stored under ambient conditions for three months. Under identical excitation, both sensors exhibited comparable output waveforms, with no significant performance degradation observed in the three‐month aged device (Figure S6, Supporting Information). This confirms the sensor's excellent stability and its suitability for long‐term monitoring applications. Finally, to simulate real‐world skin conditions, we compared the pulse detection waveforms in the states of dry skin and simulated sweating. (Figure S7, Supporting Information). Consistent with the humidity tests, sweat had no noticeable impact on the PENG sensor's output, further proving its reliability in practical applications.

A series of characterizations were also conducted on the high‐precision PPG sensor. First, tests were performed on different subjects, with one group consisting of participants of different genders. It was found that the amplitude of the pulse wave was unaffected by gender, whereas female subjects exhibited slightly higher pulse frequencies than male subjects (Figure S8a, Supporting Information). A second group compared subjects of different body weights, and similarly, the pulse wave amplitudes remained nearly identical, while the heart rate of subjects with higher body weight was slightly elevated compared to those with normal weight (Figure S8b, Supporting Information). Next, similar to PENG sensors, the effect of contact angle between the PPG sensor and skin on pulse wave signals was examined (Figure S9, Supporting Information). Measurements from the same subject at different contact angles revealed that when the PPG sensor was not properly aligned with the skin (i.e., angled contact), the pulse wave amplitude decreased significantly. This finding reinforces the necessity of a clip mechanism to ensure proper alignment by simulating finger pressure. Additionally, the influence of ambient light on PPG sensor output was investigated (Figure S10, Supporting Information). Experiments confirmed that darker environments enhance the PPG's pulse wave signal, making the system particularly suitable for nocturnal sleep monitoring. Furthermore, the SpO₂ measurement function was characterized. A continuous 5‐min simultaneous recording of SpO₂ and pulse wave curves from one subject was performed (Figure S11, Supporting Information). During the test, occlusion of the brachial artery was applied to temporarily restrict blood flow. Observations showed that after flow restriction, SpO₂ first exhibited a slow rise (values exceeding 100% indicated abnormal conditions) followed by a rapid decline, while pulse wave amplitude dropped sharply and eventually disappeared as pressure increased. Upon releasing the occlusion, both SpO₂ and pulse waves quickly recovered, demonstrating the PPG sensor's reliability in continuous SpO₂ and heart rate monitoring.

### 2.3 Mechanism of SAS and Characteristics of Physiological Signals Change

**Figure 3:**
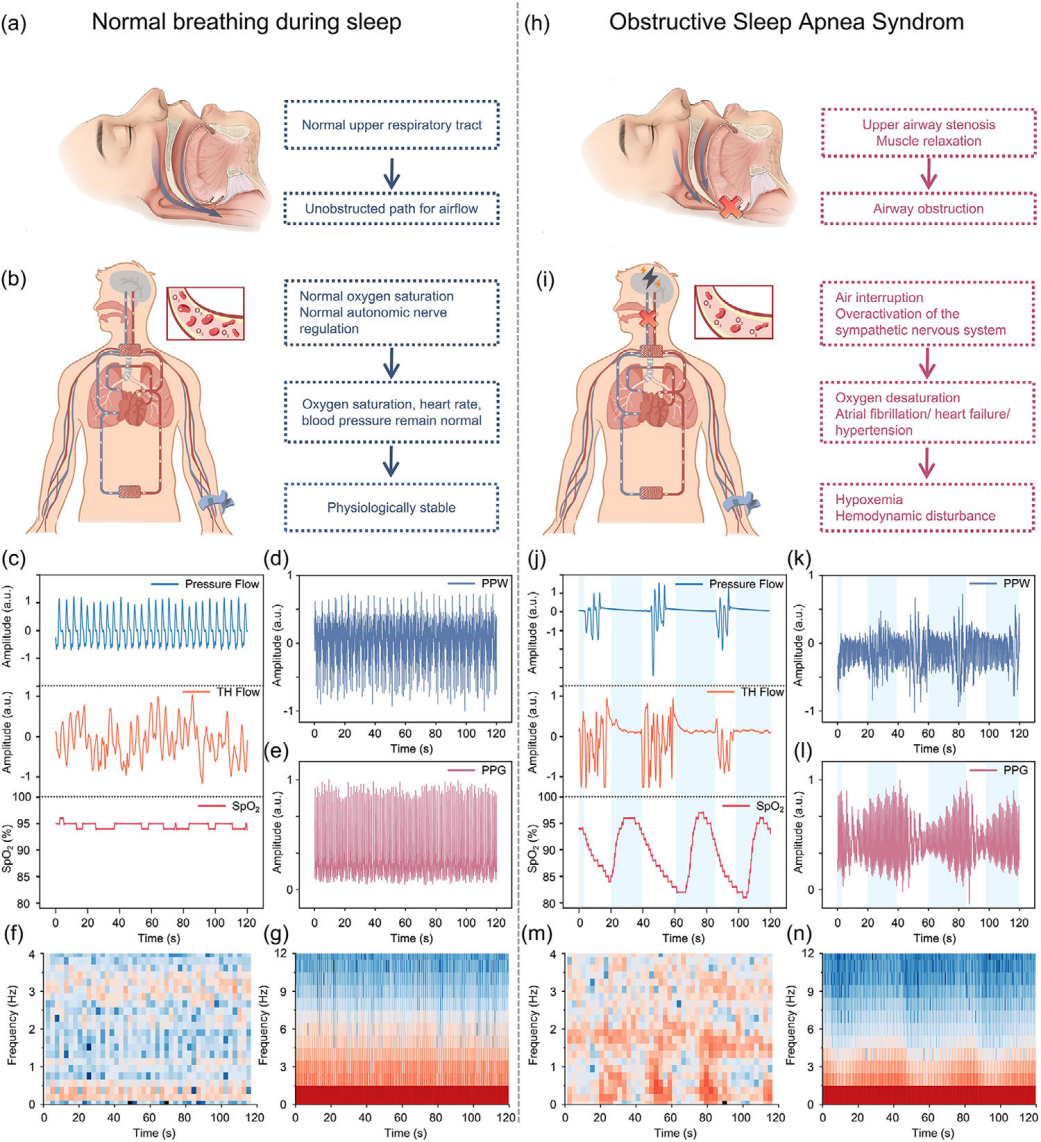
Mechanism of OSAS and Changes in Respiration and Pulse‐Related Signals. a) State of the airway during normal breathing. b) State of the circulatory system during normal breathing. c) Nasal pressure airflow signal, thermistor flow signal, and oxygen saturation signal monitored by PSG during normal breathing. d) PPW signal during normal breathing. e) PPG signal during normal breathing. f) Time‐frequency variation diagram of PPW waveform during normal breathing. g) Time‐frequency variation diagram of PPG waveform during normal breathing. h) Changes in the airway during OSAS. i) Changes in the circulatory system during OSAS. j) Changes in nasal pressure airflow signal, thermistor signal, and oxygen saturation signal monitored by PSG during OSAS. k) Changes in PPW signal during OSAS. l) Changes in PPG signal during OSAS. m) Time‐frequency variation diagram of PPG waveform during OSAS. n) Time‐frequency variation diagram of PPG waveform during OSAS.

Under normal sleep breathing conditions, as depicted in **Figure** **3**a,b, the airway remains unobstructed, and the circulatory system exhibits stable hemodynamics. Various physiological signals recorded through PSG show characteristic patterns (Figure 3c): nasal pressure airflow maintains stable amplitude with a 3–5 s cycle period, thermosensitive signals show regular fluctuations synchronous with breathing, and SpO₂ remains stable at 95–99% with minimal variation (<2%). The fingerprint PENG placed on the wrist converts the subtle vibrations of the radial artery into electrical signals, as shown in Figure 3d. Simultaneously, the PPG signal exhibits periodic undulations, with typical biphasic waveform characteristics, including the primary and dicrotic waves, as shown in Figure 3e. The amplitude of the waveform remains stable, with a variation coefficient of <10%. Time‐frequency feature analysis was performed on the PPW and PPG waveforms using the short‐time Fourier transform method. Figure 3f,g shows the spectral analysis of PPW and PPG during normal sleep breathing, revealing relatively stable time‐frequency characteristics.

As shown in Figure 3h, taking the mechanism of obstructive sleep apnea as an example, airway collapse and relaxation of the respiratory muscles lead to airflow obstruction. This obstruction causes a sharp reduction in oxygen intake, which in turn triggers a drop in blood oxygen levels, stimulating chemoreceptors and leading to excessive activation of the sympathetic nervous system. This autonomic dysregulation results in increased peripheral vascular resistance, elevated blood pressure, and altered heart rate variability. The intermittent hypoxia and reoxygenation cycles lead to oxidative stress and systemic inflammation, which further contribute to endothelial dysfunction. These pathophysiological changes manifest in the pulse wave and photoplethysmography signals, where mechanical and optical properties of the vasculature are altered, reflecting the underlying hemodynamic disturbances. This cascade of events may result in the onset of cardiovascular diseases, such as atrial fibrillation, heart failure, and hypertension, along with systemic hemodynamic imbalance, as depicted in Figure 3i.

Subsequently, a series of significant changes in physiological signals are triggered, as depicted in Figure 3j. The nasal pressure airflow signal recorded by PSG exhibits a marked reduction in waveform amplitude (>90%) or complete disappearance during the apnea event, while thermosensitive signals show minimal fluctuation. Blood oxygen saturation (SpO₂) starts to gradually decrease 10–20 s after the onset of apnea, and in severe cases, SpO₂ can drop below 90%. Figures S2 and S3 (Supporting Information) demonstrate two distinct patterns in thoracoabdominal pressure signals: During obstructive sleep apnea, thoracic and abdominal signals show increased synchronous oscillations, indicating persistent respiratory effort despite airflow obstruction. In contrast, central sleep apnea exhibits minimal to no oscillation in thoracoabdominal signals. Additionally, wrist‐mounted PENG, due to its superior mechanical‐to‐electrical conversion characteristics, detects minute changes in radial artery vibrations during apnea events (Figure 3k), with output amplitude decreasing by over 50%. Previous studies have shown that during sleep apnea events, PPG waveforms typically exhibit characteristic changes including reduced amplitude (30–50%), delayed dicrotic waves, and increased pulse transit time (PTT).^[^ ^43^, ^44^, ^45^, ^46^, ^47^ ^]^ In our monitoring results, as shown in Figure 3l, the PPG signal demonstrates decreased amplitude with altered baseline fluctuations and less distinct wave characteristics during apnea episodes. Time‐frequency analysis during apnea (Figure 3m,n) reveals distinct changes in both signals' spectral characteristics. The PPG frequency distribution shows higher intensity and non‐uniform patterns during apnea compared to normal breathing, with significant alterations in both time‐domain measures (increased SDNN) and frequency‐domain measures (elevated LF/HF ratio), reflecting autonomic nervous system modulation. The spectral analysis also reveals marked changes in signal characteristics between normal and apnea states across different frequency bands. In the later stages of apnea, compensatory mechanisms trigger vascular constriction and dilation, causing transient fluctuations in PPW signal amplitude, with the spectral characteristics displaying a multi‐peak distribution.^[^ ^48^, ^49^ ^]^ As the apnea event resolves, the signals gradually return to baseline levels.

Based on their distinctive signal characteristics, both wrist‐based PPG and PENG sensors show promise for sleep apnea detection. PPG, through its ability to capture both hemodynamic changes and blood oxygen saturation, provides multi‐dimensional physiological information. Specifically, PPG enables comprehensive analysis of the radial artery pulse waveform—including amplitude, PTT, and dicrotic wave features—along with rich HRV indicators and crucial blood oxygen levels that directly indicate apneic events. Meanwhile, PENG, with its exceptional piezoelectric response and high mechanical sensitivity, excels at capturing subtle mechanical characteristics like pulse waves and vascular pressure changes. It identifies apnea events by analyzing the time‐frequency characteristics of radial artery micro‐vibration signals, including amplitude attenuation, spectral energy shifts, and signal entropy changes. The synergistic integration of these two sensing modalities on the wrist offers significant complementary advantages: PPG provides comprehensive cardiovascular and blood oxygen information, while PENG delivers precise detection of subtle mechanical signals related to respiration and vascular dynamics. These complementary characteristics form a solid theoretical foundation for developing sleep apnea detection algorithms based on wrist‐mounted dual‐modal sensing. By combining the multi‐dimensional features of PPG with the fine mechanical characteristics detected by PENG, and utilizing advanced signal processing and machine learning methods, it is expected to significantly improve the accuracy, reliability, and real‐time performance of sleep apnea detection, while ensuring a comfortable user experience.

### 2.4 Deep Learning‐Based Intelligent Diagnosis Model for SAS

**Figure 4:**
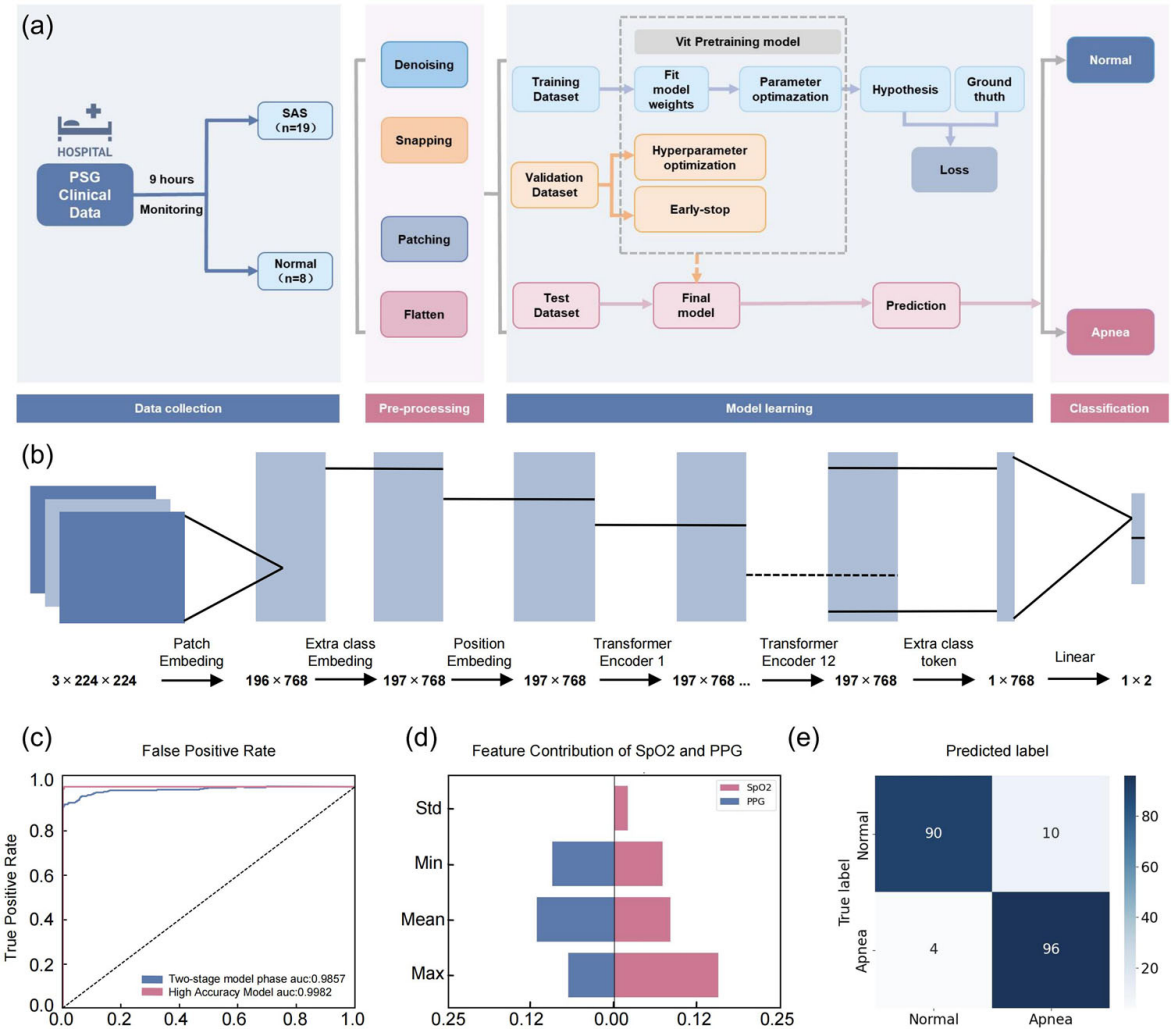
ViT Model based Sleep Apnea Detection and Performance Analysis. a) Workflow for sleep apnea classification. b) ViT Model architecture. c) ROC curve. d) Feature contribution of SpO₂ and PPG signals. e) ViT Model Confusion matrix.

Here, we explored four different modeling approaches of machine learning algorithms: Logistic Regression (LR), Support Vector Machine (SVM), eXtreme Gradient Boosting (XGBoost), and Vision Transformer (ViT). Though the original dataset comprised various sensor signals across multiple dimensions, including PPW, SpO₂, PPG, ECG, EEG, airflow signals, and 24 other parameters, during the data modeling process, only PPG and SpO₂ signals were used for modeling based on the labeled data. The data used in this work is based on the PSG laboratory database at Beijing Hospital. This database contains patient admission data from the PSG laboratory between June 1, 2024, and September 25, 2024. To minimize biases due to missing data or motion artifacts, the training data for High Accuracy Model (PPG) is extracted in the following ways: data segments with missing or invalid parameters (such as ECG lead disconnections or respiratory mask detachment) lasting more than 20 s were excluded during data collection. Specifically, each patient's PSG data was divided into segments at 2‐min long. After excluding segments with missing or corrupted data, the sleep apnea dataset included 3650 valid data segments. Additionally, the normal dataset was randomly selected from PSG recordings of normal individuals without sleep apnea, yielding 3,650 2‐min data segments (**Figure** **4**a). Each data segment was labeled as “with sleep apnea” or “without sleep apnea” based on PSG results.

The dataset was randomly divided into training, validation, and test in a ten‐fold cross‐validation way (training: validation: testing = 7:2:1), in order to avoid overfitting while improving generalization (Figure 4b). In the training subset of sleep apnea patients and normal people, we first applied univariate logistic regression to identify independent variables associated with the outcome. To further interpret the significance of low‐dimensional statistical features, we utilized Shapley values, computed using the SHapley Additive exPlanations (SHAP) method. The results (Figure 4d) indicated that the four statistical features of blood oxygen level (mean, max, min, std) and the four statistical features of peak‐to‐peak distance (mean, max, min, std) contributed to the classification outcome. However, these low‐dimensional statistical features exhibited limited effectiveness in accurately identifying SAS. This observation aligns with existing research, which suggests that while oxygen desaturation levels and heart rate variability may exhibit correlation with SAS severity, directly using low‐dimensional statistical features yields suboptimal performance in detection.

To address this limitation, we propose the use of deep learning models to extract and represent relevant information in a high‐dimensional space. Specifically, we employ the ViT model, which excels at handling complex data by utilizing self‐attention mechanisms. These mechanisms enable the ViT to capture intricate spatial and temporal dependencies within the sensor signals, such as PPG, SpO₂, and PPW signals, which are critical for sleep apnea detection. The ViT model processes these input signals by dividing them into patches and learning relationships between them. These patches are enhanced with positional embeddings and additional class embeddings, allowing the model to effectively capture both spatial and temporal dynamics. The feature extraction process in the ViT model captures subtle variations in the physiological signals, including changes in blood oxygen levels, pulse wave characteristics, and mechanical vibrations in the blood vessels. This high‐dimensional representation of features helps the model identify complex patterns that are crucial for distinguishing between sleep apnea and normal sleep, which traditional low‐dimensional methods may miss.

To evaluate the model's performance comprehensively, we utilized multiple metrics. One key component of our evaluation framework is the confusion matrix (Figure 4e), which provides insights into accuracy (ACC), sensitivity (SEN), and specificity (SPE). Additionally, we used Receiver Operating Characteristic (ROC) curves to visualize the trade‐off between sensitivity and specificity across different classification thresholds. The Area Under the Curve (AUC) metric quantifies the model's overall discriminative capability, with values ranging from 0 to 1, where a value of 1 indicates perfect classification. The AUC of the ROC curve serves as the primary performance metric for assessing our model's effectiveness. Statistical analysis and modeling were implemented using Python (version 3.10), with a significance level set at a two‐tailed P‐value <0.05. As shown in the figure, the high‐dimensional representations extracted by the ViT model for SAS‐related features exhibited significantly superior performance in SAS discrimination, achieving a substantial increase in classification accuracy from ≈80% in existing literature (based on HRV, blood oxygen, etc.) to ≈95%.

The performance metrics of conventional machine learning models (LR, SVM, and XGBoost) detailed in Tables S2–S4 (Supporting Information) demonstrate consistently lower detection accuracy compared to the Vision Transformer model across all evaluation parameters. The ViT model demonstrated the best performance in distinguishing sleep apnea patients from normal individuals or normal sleep segments. As shown in **Table** **1**, the high‐accuracy model based on PPG, blood oxygen (SpO₂), and hypoxic burden (HB) achieved a specificity of 0.9973, sensitivity of 0.9945, accuracy of 0.9959, and an AUC of 0.9982 (Figure 4c). These results indicate that the high‐accuracy model can effectively distinguish between normal and abnormal signals with extremely high precision, almost without false positives or false negatives, making it a highly reliable diagnostic tool. The highly sensitive model (PPW), which classifies using only PPW signals, achieved a sensitivity of 0.9341, indicating strong detection capability for abnormal signals, but with lower specificity of 0.5417 and an overall accuracy of 0.6857. Therefore, the Two‐stage model (PPW + PPG) combines the advantages of the piezoelectric sensor and the high‐accuracy model. In the first step, the piezoelectric sensor quickly screens for suspected abnormal signals, and in the second step, the high‐accuracy model further confirms these signals. This two‐step design significantly reduces overall energy consumption while maintaining high classification performance, achieving a specificity of 0.9635, sensitivity of 0.9251, accuracy of 0.9495, and an AUC of 0.9857. This demonstrates that the model can effectively balance performance and energy consumption in resource‐constrained environments, making it a highly practical diagnostic solution.

**Table 1:** Detection Performance of ViT model for SAS Detection.

Prediction model	Specificity	sensitivity	Accuracy
High accuracy model (PPG)	0.9973	0.9945	0.9959
Highly sensitive model (PPW)	0.5417	0.9341	0.6857
Two‐stage model (PPW+PPG)	0.9635	0.9251	0.9495

### 2.5 The Application of Dual‐Modal Wearable SAS Monitoring System

**Figure 5:**
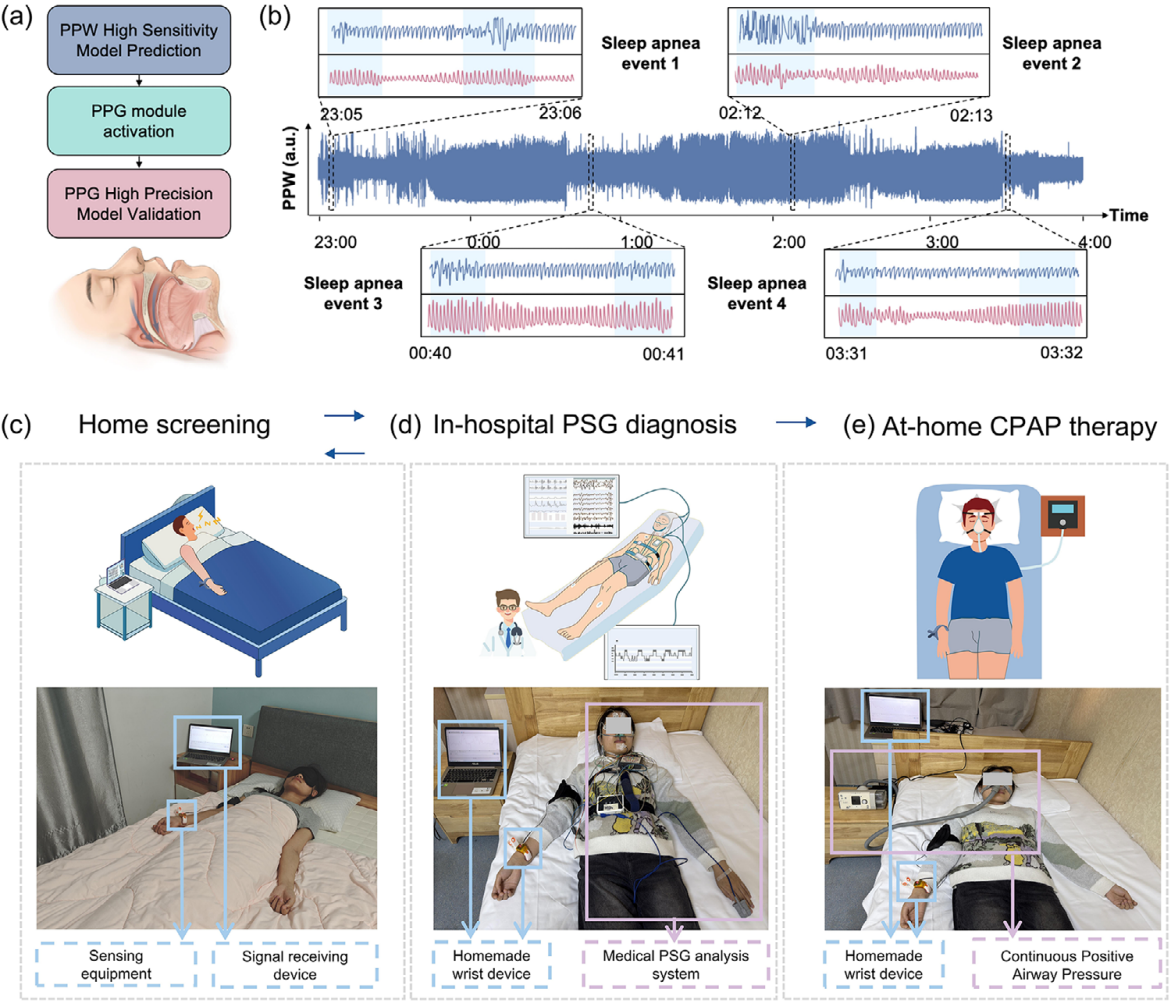
The application of dual‐modal wearable SAS monitoring system in various scenarios. a) Two‐stage detection strategy process of dual‐modal monitoring system, illustrating how PPW signals trigger PPG activation and how combined data flows through the deep learning model for final classification. b) Continuous 5‐h PPW data from a clinical measurement of a patient with sleep apnea, along with the corresponding PPW and PPG data during episodes of sleep apnea, highlighting characteristic signal changes that indicate respiratory events. c) The monitoring system used for suspicious SAS screening in home scenario, showing how the device interfaces with mobile applications for non‐invasive preliminary screening. d) The monitoring system used as a pre‐tool for PSG inspection in hospital scenario, demonstrating how it can efficiently identify high‐risk patients and optimize healthcare resource allocation. e) The monitoring system used to assess therapeutic effectiveness in home CPAP treatment setting, illustrating its role in continuous monitoring of treatment outcomes and patient compliance.

Building on the dual‐modal sensing system and two‐stage detection strategy developed in this study, we propose a real‐time sleep apnea detection solution that leverages the low‐power advantages of piezoelectric sensors and the high‐precision characteristics of PPG sensors, enabling long‐term monitoring with both energy efficiency and accuracy. In practical applications, the monitoring device is secured to the user's wrist using a C‐shaped clamp structure, ensuring stable contact with the skin via a fingertip‐like structure. The system operates in two distinct stages, as shown in **Figure** **5**a. The first stage is the continuous monitoring phase using the piezoelectric sensor. Due to the low‐power advantage of the piezoelectric sensor, the system is capable of continuously collecting pulse wave signals for 24 h. The device's embedded microcontroller processes the PPW signals in real time and triggers the second‐stage detection when a suspicious apnea event (sensitivity: 0.9341) is detected. The second stage involves precise diagnostic detection using the PPG sensor. Once a warning is triggered by the piezoelectric sensor, the system activates the PPG module for a 2‐min high‐precision detection, and the data is then input into a pre‐trained high‐accuracy model (specificity: 0.9973, sensitivity: 0.9945) for analysis to confirm whether an apnea event has occurred. This two‐stage detection strategy offers significant advantages: first, continuous pre‐screening by the piezoelectric sensor allows for the timely detection of potential apnea events; second, the PPG module, which consumes higher power, is only activated when necessary, significantly reducing overall system energy consumption and enhancing device battery life; and finally, the verification provided by the high‐accuracy model ensures the reliability of the detection results (overall accuracy: 0.9495).

The two‐stage detection strategy of the dual‐modal wearable system is illustrated in Figure S12 (Supporting Information). In this framework, PENG data is first transmitted via Bluetooth to the host computer, where the high‐sensitivity model performs an initial prediction. If the output result of the model is “Yes”, an instruction is sent to the MCU via Bluetooth to activate the PPG module. The PPG data is then sent back to the host for evaluation by the high‐accuracy model. A sleep apnea event is confirmed if the model result continues to output “Yes”, prompting data storage and an alert. If the output result of any model is “No” or the two‐stage process is completed, the system proceeds to the next cycle. Figure 5b displays partial overnight sleep data from volunteer subjects collected using our two‐stage acquisition strategy, including segments showing abnormal apnea events.

This low‐power, non‐invasive, wearable, and high‐precision detection device spans the entire process of SAS screening, diagnosis, and treatment, making it suitable for both home and hospital monitoring scenarios: 1) Home Monitoring (Figure 5c): The core goal of home monitoring is to perform initial screening for potential SAS patients using non‐invasive methods, particularly targeting individuals exhibiting symptoms such as snoring, breathing pauses, or daytime sleepiness. After the device is worn, the low‐power piezoelectric sensor continuously monitors pulse wave vibrations, and if any abnormal interruptions (e.g., irregular pulse wave amplitude or frequency) are detected, the PPG sensor is activated to capture dynamic changes in blood oxygen saturation and pulse wave. Data collected by the device is analyzed using artificial intelligence algorithms to automatically identify suspected apnea events, providing physicians with supporting data for further diagnosis. 2) Hospital Monitoring (Figure 5d): In hospital settings, the device serves as an auxiliary tool for SAS diagnosis and treatment, playing an essential role in both the initial screening and confirmation stages. It can be used as a precursor tool to PSG testing, rapidly identifying high‐risk patients through dynamic monitoring and reducing the testing burden for low‐risk users, thus optimizing healthcare resource allocation. 3) Continuous Health Management (Figure 5e): For patients who have been diagnosed or are undergoing treatment, the device provides continuous dynamic monitoring throughout the treatment process. Prior to treatment, it can be used to assess the frequency and progression of SAS, offering additional data support for patients who have not yet received treatment and helping physicians to devise the best treatment strategies. During treatment, for patients receiving continuous positive airway pressure (CPAP) therapy, the device can track the number of apneas and other key indicators to comprehensively evaluate treatment effectiveness.

For the power consumption characteristics of the two‐stage model, we need to emphasize that its actual average power consumption highly depends on the frequency of OSAS events and the duration of PPG sensor activation. The current model uses a fixed 2‐min PPG activation duration, which was chosen primarily to meet the need for signal integrity capture. Although a single sleep apnea event typically lasts only 10–30 s, the 2‐min monitoring window allows for a complete recording of the signal changes before, during, and after the event, including warning features before the apnea begins, signal features during the apnea, and the transitional period after normal breathing resumes. This ensures that the model receives enough data to make high‐accuracy judgments. As clinical data accumulates and the model is iteratively optimized, a foreseeable direction for technological development is to shorten the required PPG activation duration through more efficient feature extraction and signal processing algorithms. This would allow reliable judgments with a shorter signal collection window, maintaining or even improving detection accuracy, and providing significant support for the practicality of long‐term home sleep monitoring devices.

## 3 Conclusion

In summary, this study presents a significant advancement in sleep apnea syndrome monitoring through the development of a novel wrist‐worn device integrating PENG and PPG sensors. The core innovation lies in our bioinspired design approach, which incorporates a unique fingertip‐like structure mimicking traditional Chinese medicine pulse diagnosis, achieving stable sensor‐skin coupling and enhanced signals quality. The integration of PENG and PPG sensors provides complementary advantages: PENG enables continuous, low‐power monitoring through direct mechanical signals detection, while PPG offers comprehensive hemodynamic information for precise diagnosis. Our two‐stage detection strategy effectively balances power efficiency and diagnostic accuracy. The first stage utilizes PENG's low power consumption characteristics for continuous preliminary screening, achieving 93.41% sensitivity. The second stage activates PPG only when necessary, reaching 99.73% specificity and 99.45% sensitivity in precise diagnosis. This approach maintains a high overall accuracy of 94.95% while significantly reducing power consumption compared to conventional single‐modal systems. Our Vision Transformer‐based deep learning model demonstrates superior performance in sleep apnea detection, improving upon traditional methods based on low‐dimensional statistical features. The clinical applications span from home‐based screening to hospital diagnosis and treatment monitoring. In clinical environments, it serves as an efficient pre‐screening tool before PSG examinations and enables continuous assessment of therapy effectiveness for patients undergoing CPAP treatment. A limitation of this study is the lack of differentiation between the various subtypes of SAS, such as OSA and CSA. Future work will focus on algorithm refinement through large‐scale clinical trials and potential integration of additional biosensors to assess sleep quality and related health parameters. The monitoring system based on dual‐modal sensing and intelligent algorithms provides a systematic solution for home screening, clinical diagnosis, and treatment management of SAS, offering substantial clinical value in enhancing the diagnostic and therapeutic management of sleep apnea syndrome.

## 4 Experimental Section

### Device Fabrication

First, the preparation of the elastic layer consisted of two parts. The first part was a fingertip‐like hemispherical structure with a radius of 5 mm, which was made using PDMS. The second part was the elastic recovery layer, with dimensions of 20 mm × 40 mm × 2 mm, prepared using Ecoflex 30. The specific procedure is as follows: First, a PLA mold was printed using a 3D printer. Then, PDMS (with a mass ratio of base liquid to curing agent of 10:1) and Ecoflex 30 (with a mass ratio of Part A to Part B of 1:1) were prepared. After preparation, they were placed in a vacuum drying oven for 10 min to remove air. Next, the PPG module was positioned in the designed mold, and PDMS was slowly poured into the mold. The mold was then placed in a constant temperature chamber (at 70 °C) to cure for 2 h. After removal and cooling, the prepared Ecoflex 30 was poured into the mold. The mold was placed back in the constant temperature chamber for an additional hour of heating. After removal and demolding, a small amount of silicone gel was applied to the edges, and after drying, the device was completed. Next was the preparation of the Fingerprint PVDF. A silver‐coated PVDF film with a thickness of 52 µm was cut to a size of 15 mm × 15 mm. Then, an 8 µm thick PTFE tape was attached to both the upper and lower surfaces of the PVDF. The assembly was placed under a stamping machine, where a custom‐made tool was used to cut the multilayer film into a fingerprint pattern. Finally, wires were adhered to the surface of the silver electrode using the PTFE tape.

### Finite Element Analysis

The finite element analysis was performed using COMSOL Multiphysics software (COMSOL Burlington, MA, USA), employing a coupling strategy between the solid mechanics field and the electrostatic field. In the solid mechanics setup, the bottom of the arc‐shaped structure with different curvature radii was constrained as fixed, and the same constraint force of 1N was applied to the arc surface. The domain model of the fingerprint‐like structure was set as a piezoelectric material. In the electrostatic field setup, the upper surface of the fingerprint‐like structure was designated as a suspended electrode, while the lower surface was grounded. The material properties of the arc‐shaped structure were set to PDMS, and the material properties of the fingerprint‐like structure were set to PVDF. The study type was chosen as frequency domain analysis, with the frequency set to 1 Hz, and the model was completed.

### Characterization of Force‐Electricity Relationship

A digital tensile force gauge (Mark‐10) was used to apply and measure pressure. An oscilloscope (LeCroy, HDO6104) was used to measure the open‐circuit voltage of the PENG and PPG sensors, and to store the data during the force‐electricity test. An electrometer (Keithley 6517) was used to measure the current and charge of the sensors during electrical test characterization.

### Clinical Data Collection for High Accuracy Model (PPG)

The clinical data used for training the high‐accuracy model were sourced from patients undergoing PSG at Beijing Hospital, with the aim of providing high‐quality samples for model training. The inclusion criteria consisted of patients presenting with habitual or disruptive snoring, sleep‐related breathing pauses or sensations of suffocation, unexplained daytime sleepiness or lack of restful sleep, sleep‐related arrhythmias, or decreased blood oxygen saturation. The average apnea‐hypopnea index (AHI) for the patients was 24.5 (range: 0.8–82.3). The patients were divided into two groups: a normal group (AHI ≤ 5) with 8 participants, mean age 50 years (range: 40–75 years), mean BMI 18.5; and a severe OSAS group (AHI ≥ 30) with 19 participants (15 men, 4 women), mean age 45.2 years (range: 25–73 years), mean BMI 25.11 (range: 21.6–32.5). All participants underwent an average of 472.3 min of PSG monitoring (range: 452–540 min), and PPG wave and SpO₂ data were extracted from the PSG recordings. Each patient's monitoring data were segmented into 2‐min intervals. After excluding segments with missing or invalid data, the normal group and the apnea group contained 3650 segments each, for a total of 7300 data segments. This ensured that the dataset covered a broad spectrum of features, providing multidimensional input for model training. As this was a retrospective study, approval for waiver of informed consent was granted.

### Clinical Data Collection for Two‐Stage Model (PPW + PPG)

To evaluate the monitoring performance of the device in practical applications, the study participants were also sourced from patients undergoing PSG at Beijing Hospital. A total of 15 patients (11 males, 4 females) were enrolled, presenting symptoms such as habitual or disruptive snoring, sleep‐related breathing pauses or sensations of suffocation, unexplained daytime sleepiness or lack of restful sleep, sleep‐related arrhythmias, or decreased blood oxygen saturation. The mean age of the patients was 44.7 years (range: 24–68 years), with a mean BMI of 24.71 (range: 21–33.7). All patients underwent overnight PSG monitoring in the Beijing Hospital Sleep and Respiratory Monitoring Unit, with an average duration of 474.4 min (range: 454–540 min), during which device testing was also conducted.

During the testing period, PSG data were interpreted by clinical experts. Following the test, the average AHI for the patients was 23.7 (range: 0.6–71.8). During the device testing, PPG wave and SpO₂ parameters from the PSG data were extracted, while pulse wave signals from the device were also collected, specifically including PPG signals and PPW signals. The PPG signals were sampled at 256 Hz, while the PPW signals were sampled at 50 Hz. These varying sampling frequencies were selected to balance signals characteristics with power consumption, ensuring the device could accurately capture dynamic signals under low‐power conditions.

All monitoring data were divided into 2‐min intervals to ensure temporal consistency between the PSG data and the device‐acquired signals, while excluding invalid or corrupted data. Data collection strictly adhered to the principles outlined in the Declaration of Helsinki and its subsequent revisions, and was approved by the Beijing Hospital Ethics Committee (Approval No: 2024BJYYEC‐KY140‐01). All participants signed an informed consent form prior to the study, acknowledging the study's purpose, methods, potential risks, and privacy protection measures, ensuring that data collection and processing complied with ethical standards.

## Conflict of Interest

The authors declare no conflict of interest.

## Author Contributions

J.W., J.T.X., and Y. Z. contributed equally to this work. Y.Z., K.X., Z. L., and T.Z. conceptualized and adapted the topics and experiments. J.T.X., Y.Q.W., and Y.Z. performed the hardware design and sensor integration. J.W., Y.X.M., and F.D. contributed to data collection and clinical testing. J.H.X. and K.X. conducted the deep learning model development and analysis. Y.M.L., F.D., and T.Z. supervised the clinical data analysis. J.W., J.T.X., and Y.Z. completed the paper writing and was guided by Y.Z., K.X., Z.L., and T. Z.

## Supporting information



Supporting Information

## Data Availability

The data that support the findings of this study are available from the corresponding author upon reasonable request.
