# Supplementary material for: A Dual‐Modal Wearable Pulse Detection System Integrated with Deep Learning for High‐Accuracy and Low‐Power Sleep Apnea Monitoring
Source: Adv Sci (Weinh). 2025 Apr 29;12(24):2501750. doi: 10.1002/advs.202501750 (PMC12199612; doi:10.1002/advs.202501750)
Supplement: Supplementary file 1 — Supporting Information [file ADVS-12-2501750-s001.docx]

Supporting Information

Title: A dual-modal wearable pulse detection system integrated with deep learning for high-accuracy and low-power sleep apnea monitoring

*Jia Wang, Jiangtao Xue, Yang Zou*, Yuxin Ma, Junhan Xu, Yanming Li, Fei Deng, Yiqian Wang, Kai Xing*, Zhou Li*, Tong Zou**

J. Wang, T. Zou

Department of Cardiology, Beijing Hospital, National Center of Gerontology, Institute of Geriatric Medicine, Chinese Academy of Medical Sciences & Peking Union Medical College, Beijing 100730, China
E-mail: zoutong2001@163.com

J. Xue, Y. Zou, Y. Wang, Z. Li

Beijing Institute of Nanoenergy and Nanosystems, Chinese Academy of Sciences, Beijing 101400, China

E-mail: zouyang@bit.edu.cn; zli@binn.cas.cn

J. Xue, Y. Zou

School of Medical Technology, Beijing Institute of Technology, Beijing 100081, China

1. Xu, K. Xing

School of Computer Science and Suzhou Institute for Advanced Research, University of Science and Technology of China, Hefei 230026, Anhui, China

E-mail: [kxing@ustc.edu.cn](mailto:kxing@ustc.edu.cn)

Y. Ma

Department of Cardiology, Beijing Hospital, National Center of Gerontology, Institute of Geriatric Medicine, Chinese Academy of Medical Sciences & Peking University Fifth School of Clinical Medicine, Beijing 100730, China

Y Li, F Deng

Department of Pulmonary and Critical Care Medcine, Beijing Hospital, National Center of Gerontology; Institute of Geriatric Medicine, Chinese Academy of Medical Sciences, Beijing 100730, China

Z. Li

School of Nanoscience and Technology, University of Chinese Academy of Sciences, Beijing 100049, China

J. Wang, J. Xue and Y. Zou contributed equally to this work.

| 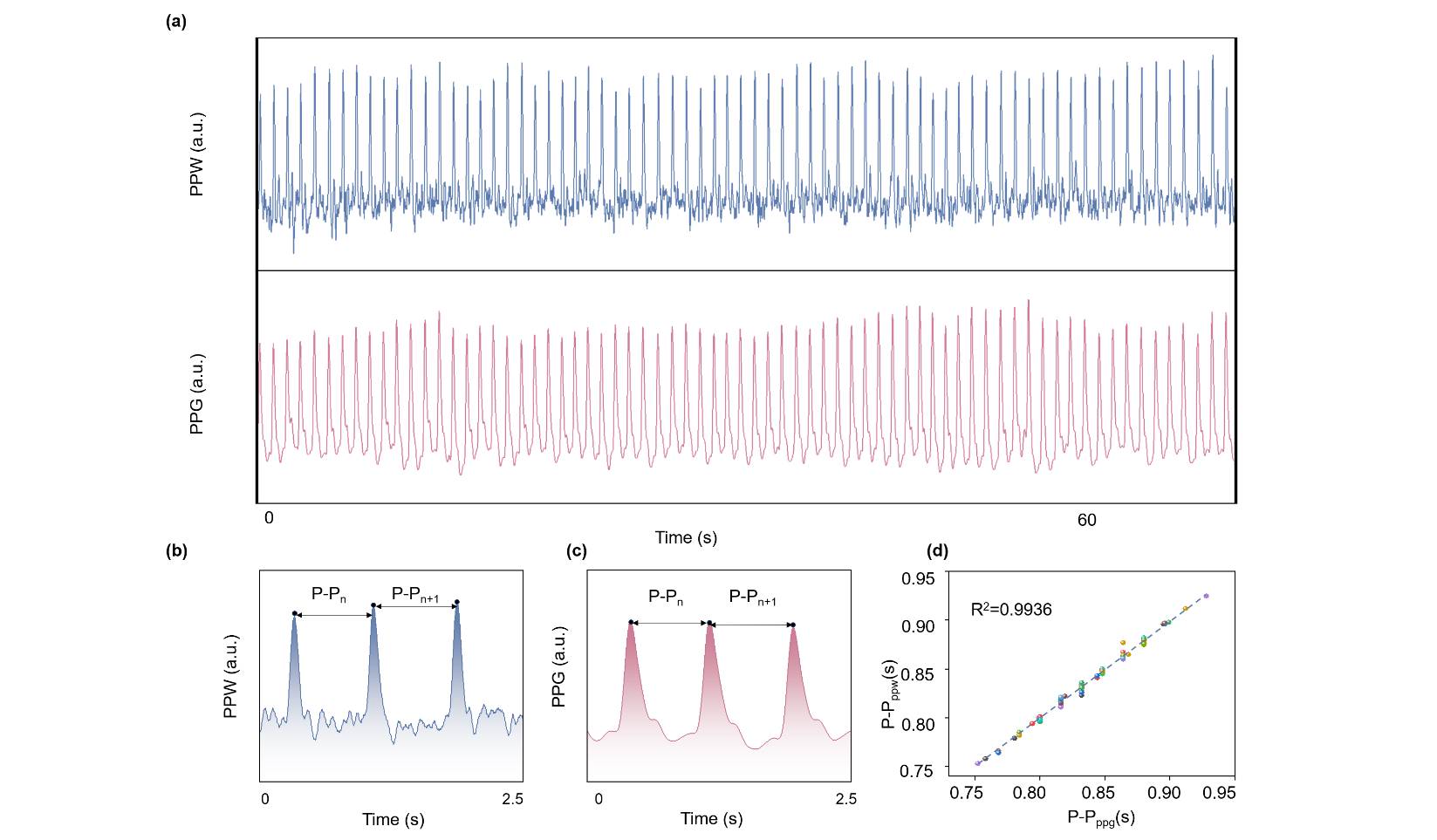 |
| --- |

**Figure S1 Comparison of pulse measurements between PPW and PPG.** (a) 1-minute pulse waveform; (b) Adjacent peak interval diagram of PPW; (c) Adjacent peak interval diagram of PPG; (d) The linear relationship of peak interval between PPW and PPG.

Figure S2. Waveform of the PPW sensing module for pulse monitoring with different curvature radius arc structures.

| **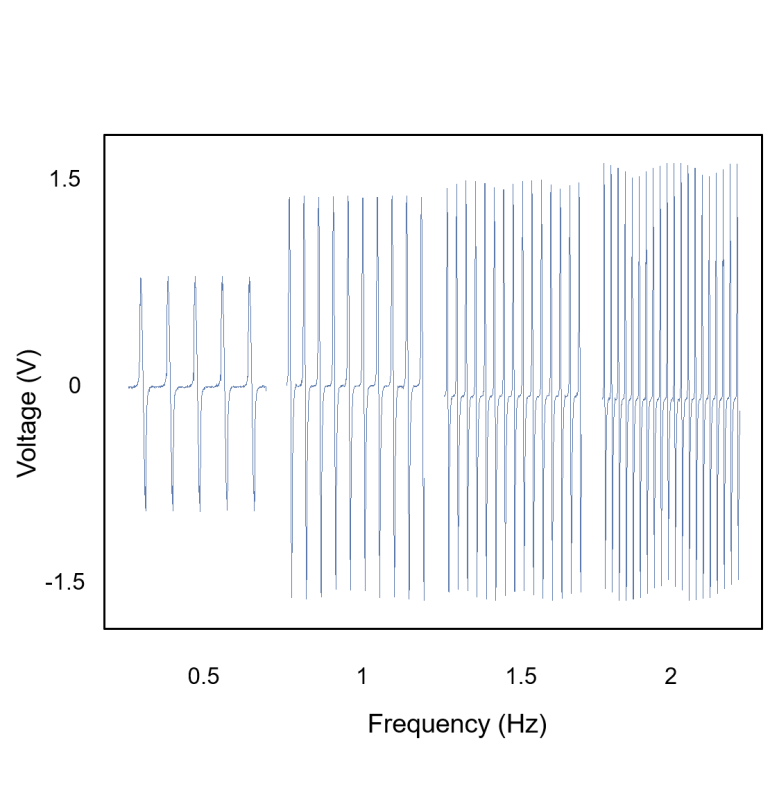** |
| --- |

Figure S3. 10-second waveforms of PENG sensors at different frequencies.

| 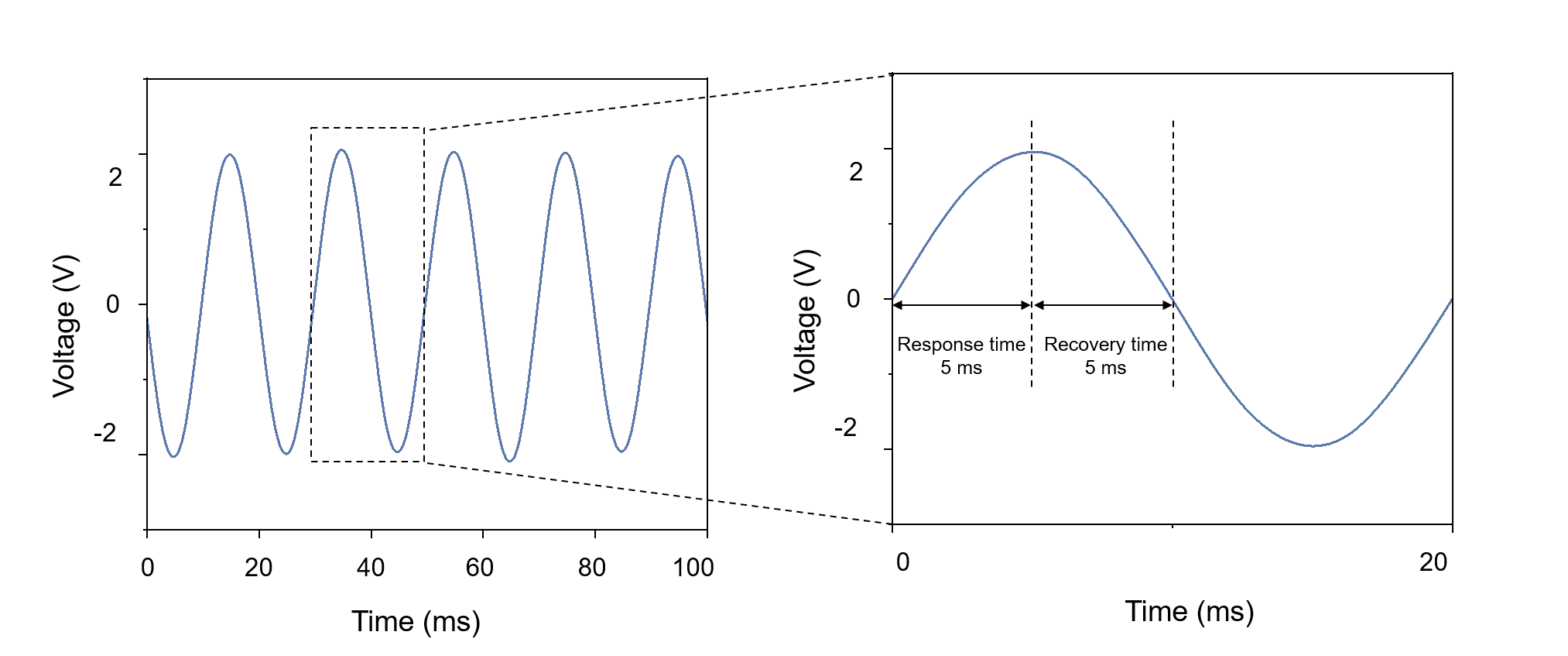 |
| --- |

Figure S4. The PENG sensor achieves a 50 ms response/recovery time under 50 Hz external vibration

| **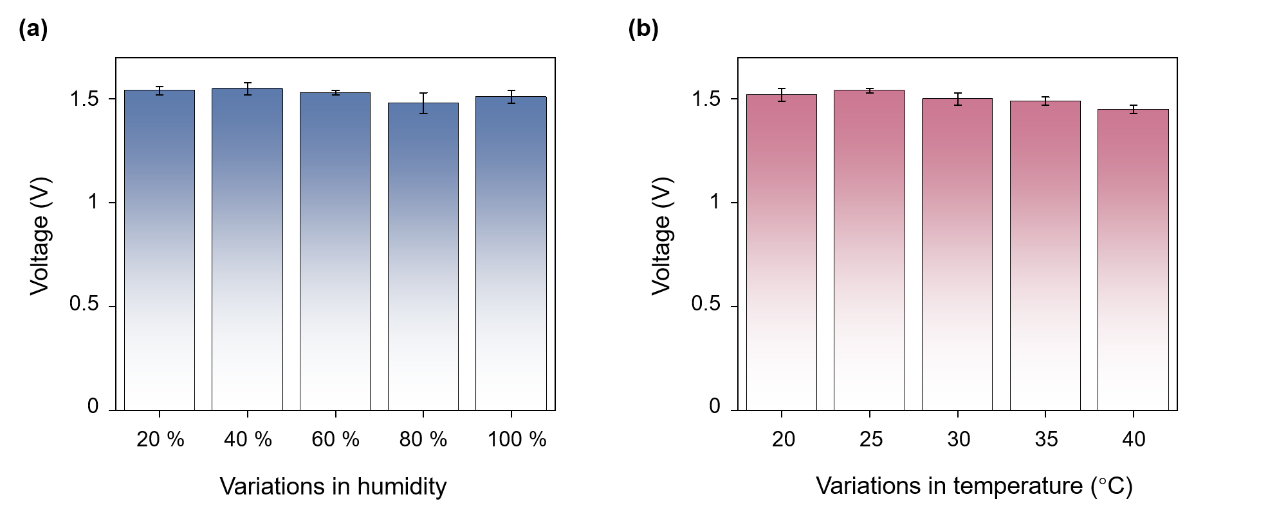** |
| --- |

Figure S5. The effect of different temperature and humidity conditions on PENG output. a) variations in humidity; b) variations in temperature.

| 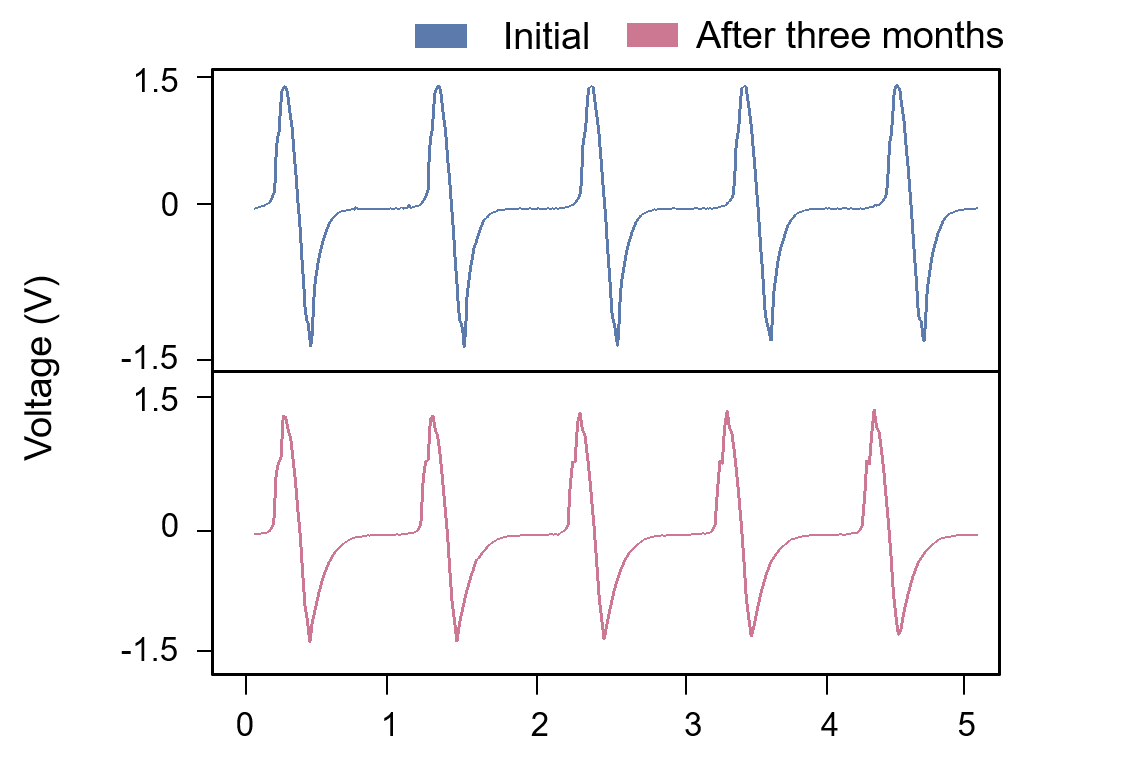 |
| --- |

Figure S6. Long-term stability assessment of PENG sensors

| 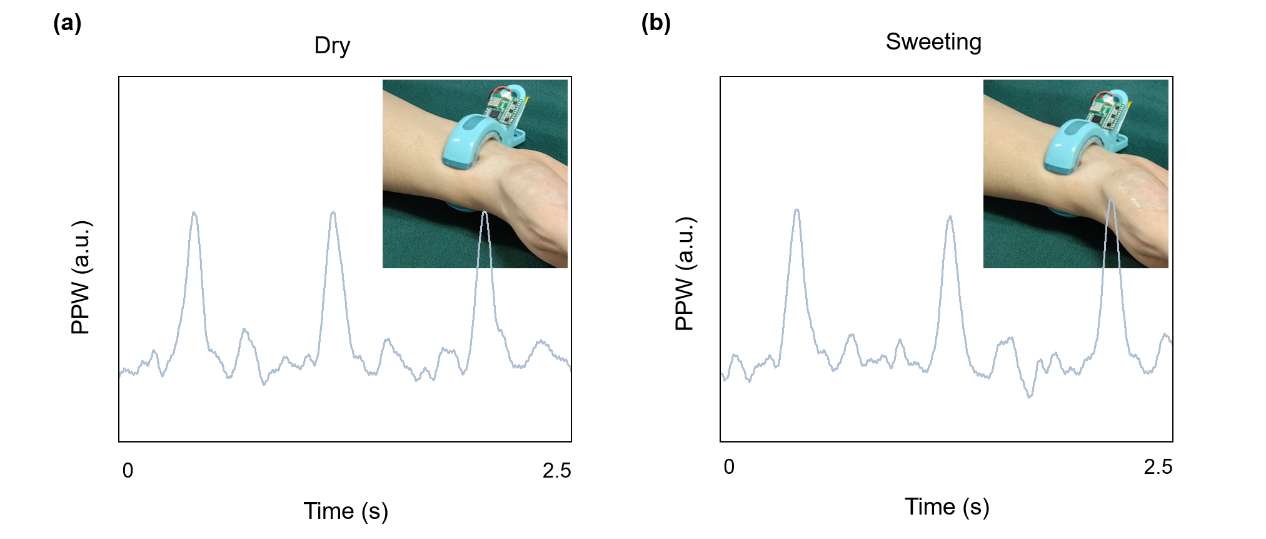 |
| --- |

Figure S7. The impact of different skin perspiration levels on PENG-based pulse wave measurement. a) Dry; b) Sweeting.

| **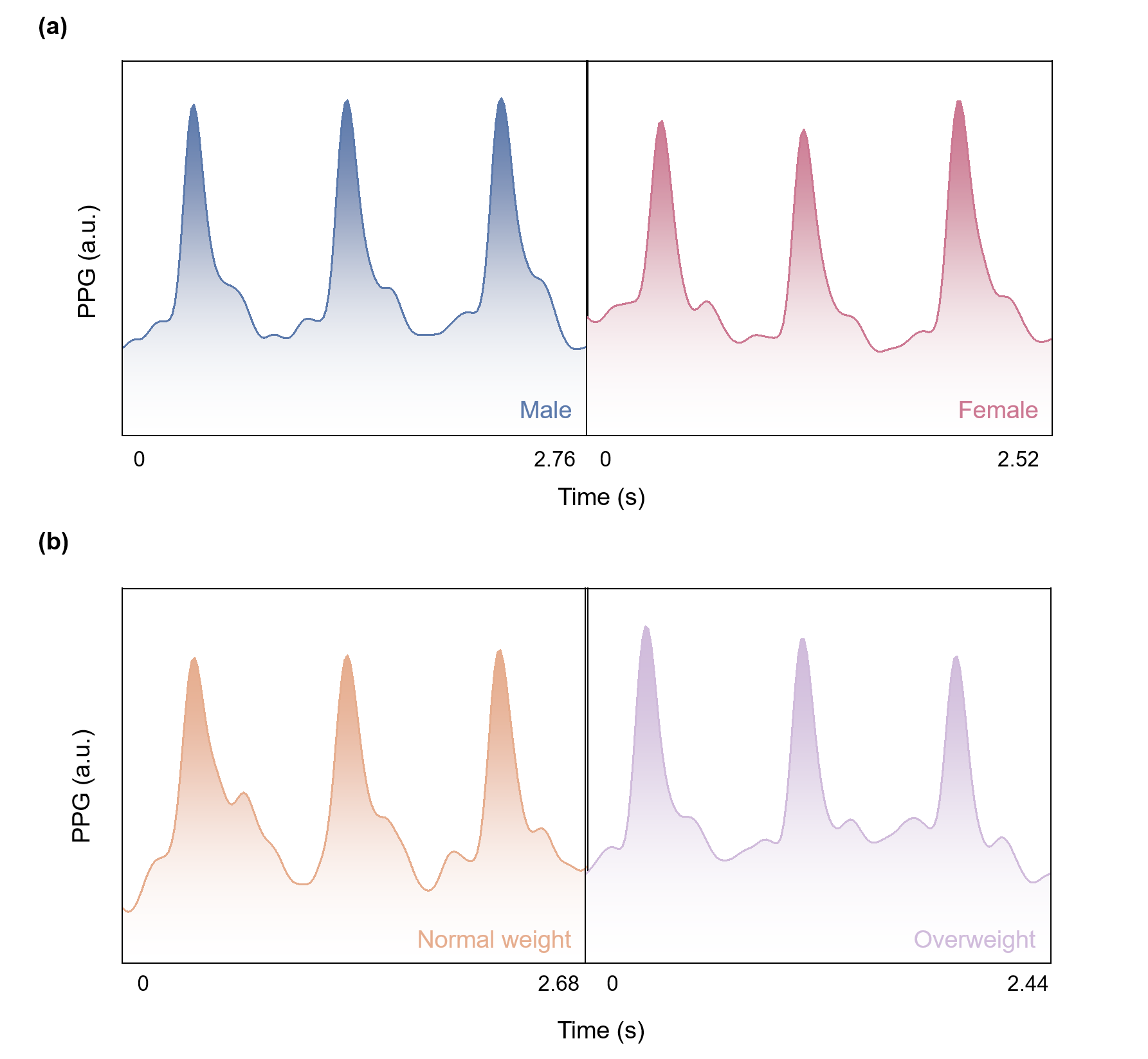** |
| --- |

**Figure S8 Pulse wave testing across different individuals based on PPG sensor.** (a) different gender; (b) different weight.

| 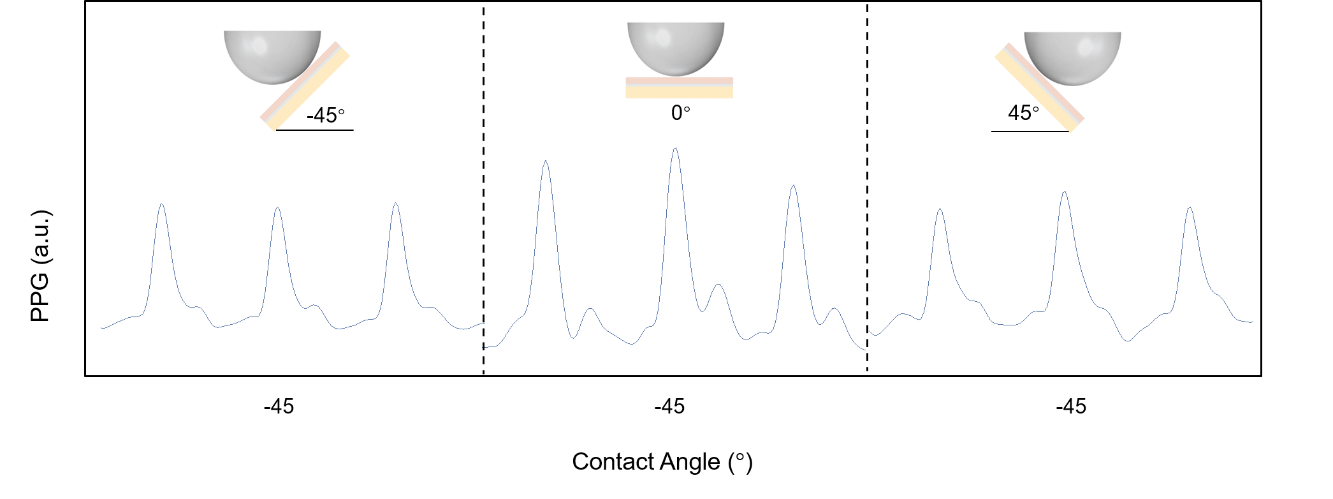 |
| --- |

Figure S9 Pulse wave testing across different contact angels based on PPG sensor.

| 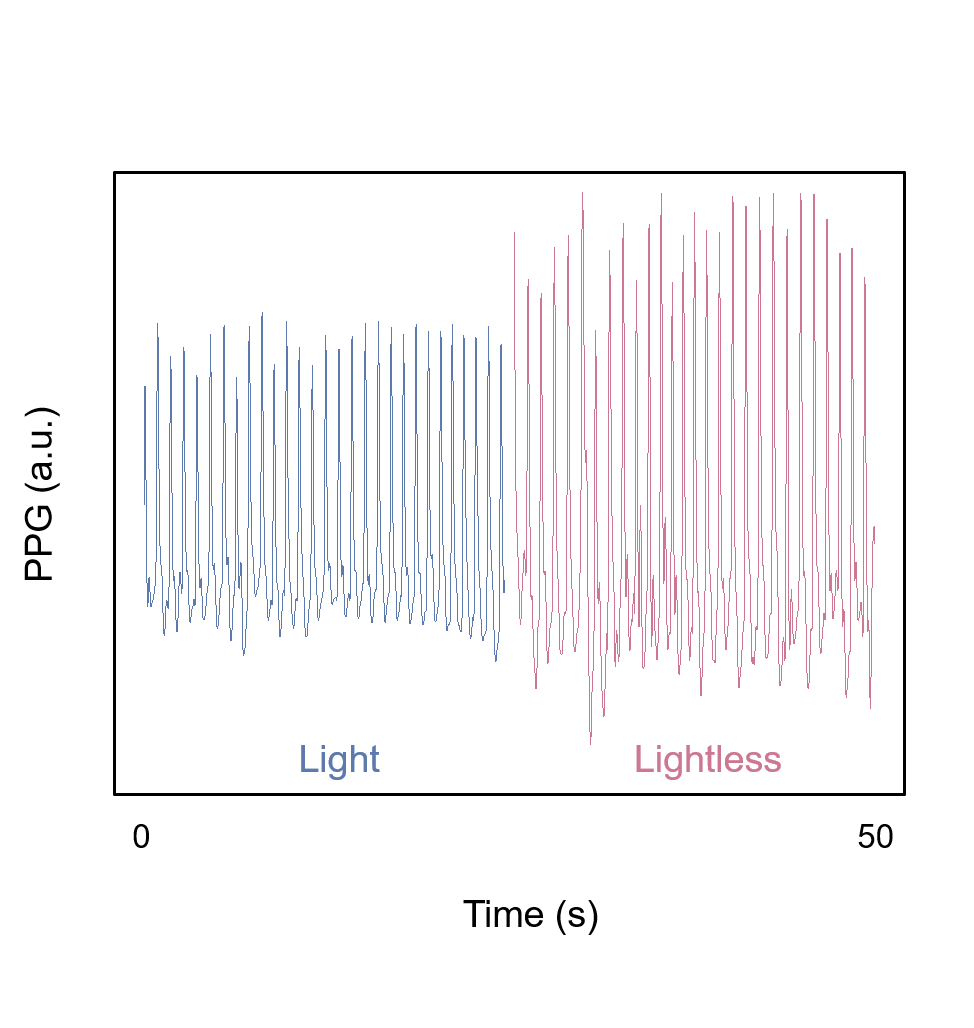 |
| --- |

Figure S10 Pulse wave testing under different light conditions based on PPG sensor.

| 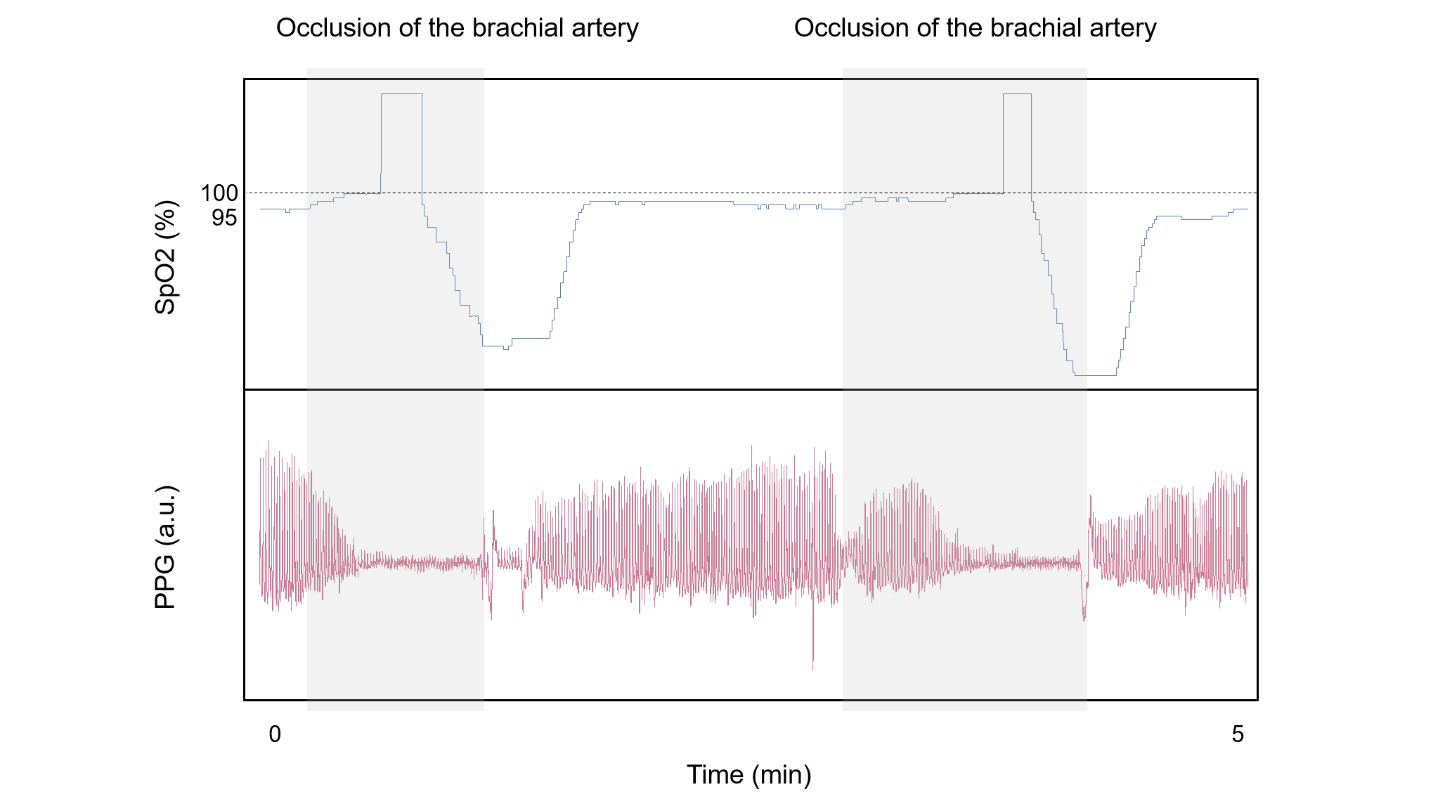 |
| --- |

Figure S11 Radial artery SpO_2_ and pulse wave testing under brachial artery blood flow Restriction.

| 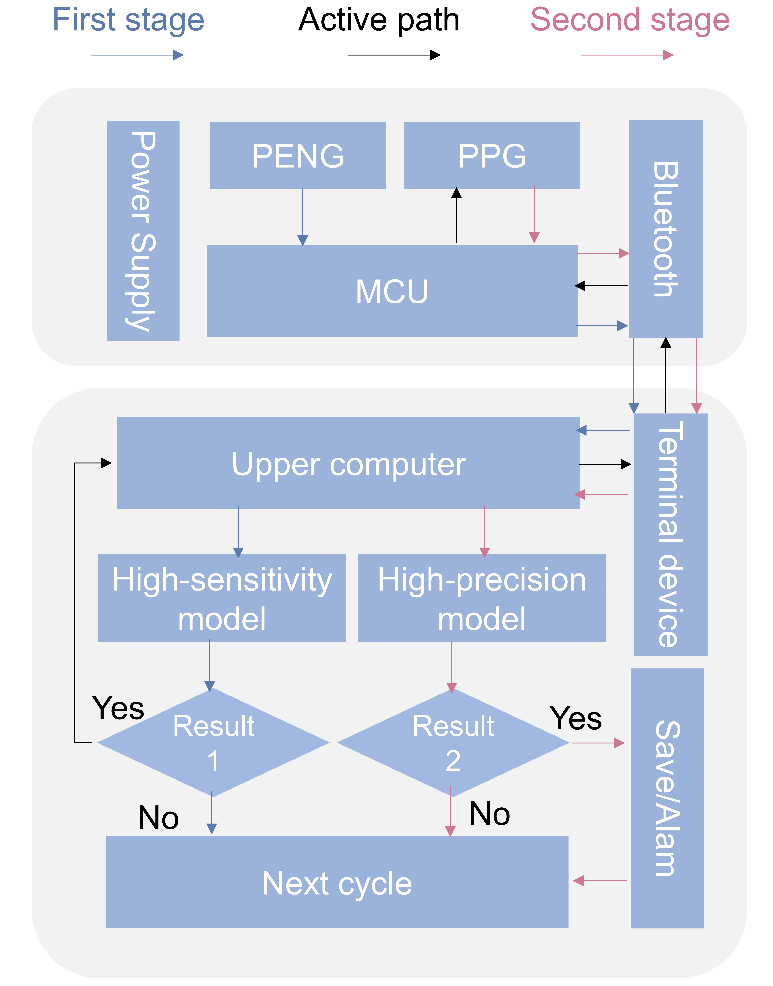 |
| --- |

Figure S12. The framework of the two-stage detection strategy of the dual-modal wearable system.

**Table S1.** Comparison of our dual-modal wearable pulse detection system with existing SAS detection methods.

| **Method** | **Detection Principle** | **Sensor Type** | **Algorithm** | **Accuracy** | **Power Consumption** | **Advantages** | **Limitations** | **Ref.** |
| --- | --- | --- | --- | --- | --- | --- | --- | --- |
| Our dual-modal system | PPW + PPG + SpO₂ | PENG + PPG (wrist-worn) | Vision Transformer | 94.95% (two-stage), 99.59% (high-accuracy) | Low (two-stage strategy) | High accuracy, low power, non-invasive, continuous monitoring | Requires further validation on larger populations | This work |
| PSG | Multiple physiological parameters | Multi-channel sensors | Expert scoring | Gold standard | High | Comprehensive evaluation, gold standard | High cost, complex setup, laboratory environment required, discomfort | [4] |
| HRV-based | Heart rate variability | ECG or PPG | Statistical analysis, SVM | 86.67% | Medium | Non-invasive, portable | Limited accuracy for mild SAS | [17] |
| PPG-based | Pulse wave & SpO₂ | PPG (wrist/finger) | Multi-task learning (1D-MMResSNet) | 95.65% | Medium-high | Good accuracy, non-invasive | High power consumption, limited monitoring time | [18] |
| Respiratory effort-based | Thoracoabdominal movement | Piezoelectric sensors | Wavelet analysis | 81.8%± 9.4% | Low | Low power, distinction between OSA and CSA | Reduced accuracy in mixed apnea | [31] |
| Acoustic-based | Respiratory sound analysis | Acoustic sensors/Microphone | Deep learning/CNN | 92.4% | Medium | Non-contact, continuous monitoring during sleep | Environmental noise interference, position-dependent accuracy | [8] |
| Oximetry-based | Blood oxygen desaturation patterns | Pulse oximeter | Deep learning models | F1 score: 0.84, ICC: 0.96 | Low-medium | Simple setup, single-channel monitoring | Delay in detection, indirect measure | [20] |
| Acoustic-based | Respiratory sound analysis | PENG attached to throat | CNN with GASF image transformation | 99.0% | Low (self-powered) | Non-contact, self-powered, high accuracy, early screening capability | Limited clinical validation, position-dependent | [30] |

Table S2. Detection Performance of LR model for SAS Detection.

| Prediction model | Specificity | Sensitivity | Accuracy |
| --- | --- | --- | --- |
| High accuracy model (PPG) | 0.5616 | 0.7178 | 0.6397 |
| Highly sensitive model (PPW) | 0.4566 | 0.4611 | 0.4582 |
| Two-stage model (PPW+PPG) | 0.7708 | 0.4132 | 0.6396 |

Table S3. Detection Performance of SVM model for SAS Detection.

| Prediction model | Specificity | Sensitivity | Accuracy |
| --- | --- | --- | --- |
| High accuracy model (PPG) | 0.9315 | 0.9452 | 0.9384 |
| Highly sensitive model (PPW) | 0.3958 | 0.6168 | 0.4769 |
| Two-stage model (PPW+PPG) | 0.9583 | 0.6018 | 0.8275 |

Table S4. Detection Performance of XGBoost model for SAS Detection.

| Prediction model | Specificity | Sensitivity | Accuracy |
| --- | --- | --- | --- |
| High accuracy model (PPG) | 0.9671 | 0.9452 | 0.9562 |
| Highly sensitive model (PPW) | 0.3663 | 0.8204 | 0.5330 |
| Two-stage model (PPW+PPG) | 0.9462 | 0.8144 | 0.8978 |
